# Naturally derived Heme-Oxygenase 1 inducers attenuate inflammatory responses in human dendritic cells and T cells: relevance for psoriasis treatment

**DOI:** 10.1038/s41598-018-28488-6

**Published:** 2018-07-06

**Authors:** Nicole K. Campbell, Hannah K. Fitzgerald, Anna Malara, Roisin Hambly, Cheryl M. Sweeney, Brian Kirby, Jean M. Fletcher, Aisling Dunne

**Affiliations:** 1 School of Biochemistry & Immunology and School of Medicine, Trinity Biomedical Sciences Institute, Trinity College Dublin, the University of Dublin, Dublin 2, Ireland; 2 Department of Dermatology, St. Vincent’s University Hospital, Dublin 4, Ireland

## Abstract

Psoriasis is a chronic autoimmune disease mediated by dysregulated immune responses in dendritic cells (DC) and T cells. The stress-response enzyme heme oxygenase-1 (HO-1) has been described as protective in animal models of psoriasis, however, implementation of HO-1-based therapies is hindered by the lack of clinically-suitable HO-1 inducers. The plant-derived polyphenols, carnosol and curcumin, have been identified as candidate HO-1 inducers however there has been little investigation into their effects on human immune cells. We demonstrate that treatment of human DC with these polyphenols limits DC maturation, reduces pro-inflammatory cytokine production, and prevents induction of allospecific T cell responses, in a manner partially dependent on carbon monoxide (CO). We also characterised their effects in *ex-vivo* psoriasis PBMC and report that curcumin, but not carnosol, strongly reduces T cell proliferation and cytokine poly-functionality, with reduced expression of psoriatic cytokines IFNγ, IL-17, GM-CSF and IL-22. This study therefore supports reports highlighting the therapeutic potential of curcumin in psoriasis by providing insight into its immunological effects on healthy human DC and psoriasis PBMC. We also demonstrate, for the first time, the anti-inflammatory effects of carnosol in human immune cells.

## Introduction

Psoriasis is a chronic autoimmune disease of the skin affecting 2–3% of the population, which manifests as red, scaly plaques with associated pruritus and pain^1^. It is characterised by excessive keratinocyte proliferation as well as extensive infiltration and activation of immune cells^2^. Although the causes of psoriasis are incompletely understood, important roles for dendritic cells (DC) and T cells in the pathophysiology of psoriasis have been identified in recent years, and particular emphasis has been placed on the Th17 axis cytokines, IL-23, IL-17 and IL-22^3–6^.

Heme oxygenase-1 (HO-1) is a stress-inducible enzyme which catalyses the conversion of heme to the linear tetrapyrroles biliverdin (BV) and bilirubin (BR), with the concomitant release of carbon monoxide (CO). All three of these reaction products have potent anti-inflammatory and antioxidant properties^7–9^. Previous studies have indicated that HO-1 may inhibit skin inflammation and abnormal keratinocyte proliferation in psoriasis^10^. Upregulation of HO-1 by metalloporphyrins has been demonstrated to improve symptoms of psoriasis in animal models^11–13^. Furthermore, induction of HO-1 has been associated with existing treatments for psoriasis. For example, phototherapy involving exposure to ultraviolet radiation has been shown to upregulate HO-1 expression in human skin, while the immunosuppressant dimethyl fumarate (DMF) has been identified as a potent HO-1 inducer, and at least some of its anti-inflammatory effects have been attributed to HO-1^14–16^.

At a cellular level, HO-1 has been identified as an immunomodulator in DC, an important cell type in psoriasis pathogenesis. Expression of the enzyme is associated with the maturation status of DC, with immature or tolerogenic DC expressing high levels of HO-1, while expression appears to be downregulated in mature DC^17–19^. This is supported by studies demonstrating that induction of HO-1 promotes tolerogenic DC by inhibiting their pro-inflammatory functions and maintaining them in an immature-like state^18,20^. Of note, promotion of tolerogenic DC and reduced production of the Th17-polarising cytokine, IL-23, by DC has previously been attributed to the efficacy of DMF in psoriasis^16^. While comparatively fewer studies have investigated the role of HO-1 in T cells, there have been reports that HO-1 and its reaction products can inhibit T cell proliferation^21–23^.

Despite the mounting evidence supporting upregulation of HO-1 as a treatment strategy for inflammatory diseases, translating many of these studies to the clinic has been hindered by the lack of suitable HO-1 inducers. Traditional compounds primarily include metalloporphyrins, which strongly upregulate HO-1 expression, but are also associated with significant toxicity. Therefore, there is a solid rationale to identify safer and better tolerated alternatives to currently available HO-1 inducers given its well established role as an anti-inflammatory mediator. It has been reported that the plant-derived polyphenols, carnosol and curcumin, are capable of inducing HO-1 expression^24–27^. Curcumin has previously been demonstrated to inhibit the maturation and pro-inflammatory functions of murine and human DC, and has shown efficacy in murine models of psoriasis^28–31^. Additionally, curcumin has been shown to improve psoriasis symptoms in three small clinical trials, however, there is limited insight into its effects on immune cells^32–34^. To date there are no studies investigating the anti-inflammatory effects of carnosol in human immune cells, either *in vitro* or as a treatment for psoriasis.

In this study, we sought to examine the ability of carnosol and curcumin to modulate immune responses in human DC and T cells, due to their important roles in psoriasis pathophysiology. We demonstrate that treatment with carnosol or curcumin prior to stimulation with lipopolysaccharide (LPS) limits DC maturation, reduces production of pro-inflammatory cytokines and attenuates proliferation of allogeneic T cells. We also demonstrate that these effects are at least partially mediated by the HO-1 reaction product, CO. Finally, we have examined the effects of carnosol and curcumin in peripheral blood mononuclear cells (PBMC) isolated from psoriasis patients, and report that curcumin significantly inhibits T cell proliferation, pro-inflammatory cytokine production and polyfunctionality *ex-vivo*. These findings add to the current knowledge of the anti-inflammatory and anti-psoriatic effects of curcumin and demonstrate its ability to reduce pro-inflammatory cytokine production in *ex-vivo* psoriasis patient PBMC. Additionally, our results provide evidence of a novel role for carnosol as an immunomodulator in human DC.

## Materials and Methods

### Reagents

Carnosol (from *Rosemarinus officinalis*) and curcumin (from *Curcuma longa*) were purchased from Sigma-Aldrich and dissolved in DMSO. Human hemoglobin was purchased from Sigma-Aldrich and dissolved in RPMI. Ultrapure lipopolysaccharide (LPS) was purchased from Invivogen. The HO-1 inhibitor tin-protoporphyrin IX (SnPP) was purchased from Frontier Scientific and dissolved in 50 mM TRIS buffer.

### Human blood samples

This study was approved by the research ethics committee of the School of Biochemistry and Immunology, Trinity College Dublin and was conducted in accordance with the Declaration of Helsinki. Leukocyte-enriched buffy coats from anonymous healthy donors were obtained with permission from the Irish Blood Transfusion Service (IBTS), St. James’s Hospital, Dublin. Donors provided informed written consent to the IBTS for their blood to be used for research purposes. PBMC were isolated by density gradient centrifugation (Lymphoprep; Axis-Shield poC). Cells were cultured in RPMI medium supplemented with 10% FCS, 2 mM L-glutamine, 100 U/ml penicillin and 100 µg/ml streptomycin (all Sigma Aldrich) and maintained in humidified incubators at 37 °C with 5% CO_2_.

### Psoriasis PBMC samples

Written informed consent was obtained from participants attending a specialist psoriasis out-patient clinic at St Vincent’s University Hospital, Dublin. The study received ethical approval from St. Vincent’s University Hospital Ethics and Medical Research Committee and was conducted in accordance with the Declaration of Helsinki. Blood samples were taken from consenting patients (n = 9; mean PASI 6.6 ± 3.3) and PBMC were isolated and frozen at −80 °C. PBMC were thawed and incubated at 1 × 10^6^ per ml in the presence of carnosol (5 µM), curcumin (5 µM), or a vehicle control (DMSO) for 6 hours prior to stimulation with anti-CD3 (1 µg/ml; eBioscience). After 4 days supernatants were removed for analysis of IFNγ and IL-17A concentrations by ELISA (Ready-Set-Go kit; eBioscience), and PBMC were restimulated for flow cytometry.

### Dendritic cell culture

CD14^+^ monocytes were positively selected from PBMC by magnetic sorting using a MagniSort Human CD14 Positive Selection kit (eBioscience) according to the manufacturer’s protocol. Monocyte derived DC were produced by culturing purified CD14^+^ monocytes at 1 × 10^6^ cells/ml in complete RPMI supplemented with GM-CSF (50 ng/ml) and IL-4 (40 ng/ml; both Miltenyi Biotec). On the third day of culture half the media was removed and replaced with fresh media supplemented with cytokines. After six days non-adherent and loosely adherent cells were gently removed. The purity of CD14^lo^DC-SIGN^+^ DC was assessed by flow cytometry and was routinely >98%.

DC were cultured at 1 × 10^6^ cells/ml in the presence of carnosol (2.5–10 µM), curcumin (2.5–10 µM) or vehicle control (DMSO) for 6 hours prior to stimulation with LPS (100 ng/ml). After 24 hours cell supernatants were removed for analysis of IL-12p70, IL-23p19, TNFα and IL-10 cytokine concentration by ELISA (Ready-Set-Go kits, eBioscience) and DC were either removed for analysis by flow cytometry, or used in further assays.

### DC-CD4^+^ T cell co-cultures

CD4^+^ T cells were positively selected from PBMC by magnetic sorting using a MagniSort Human CD4 T cell Positive Selection kit (eBioscience), and stained with CellTraceViolet (Life Technologies) according to the manufacturer’s instructions. CD4^+^ T cells were co-cultured with allogeneic DC that had been pre-treated with carnosol, curcumin or vehicle control and activated with LPS as previously described. Cells were co-cultured at a ratio of 1:10 DC to CD4 cells with no carnosol or curcumin present. Cells were collected after 5 days for analysis of CD4^+^ T cell proliferation by flow cytometry, and supernatants were removed for analysis of IFNγ concentration by ELISA (Ready-Set-Go kit; eBioscience).

### Western Blotting

For detection of HO-1 expression, DC or PBMC were cultured at 1 × 10^6^ cells/ml in the presence of carnosol (1–10 µM), curcumin (1–10 µM) or a vehicle control (DMSO), with or without LPS (100 ng/ml), for 24 hours. For detection of MAPK activation, DC were cultured at 1 × 10^6^ cells/ml in the presence of carnosol (10 µM), curcumin (10 µM) or DMSO, with or without SnPP (50 µM), for 6 hours prior to stimulation with LPS (100 ng/ml) for 15 minutes to 3 hours. For detection of pro-IL-1β production, DC were cultured at 1 × 10^6^ cells/ml in the presence of carnosol (10 µM), curcumin (10 µM) or DMSO for 6 hours prior to stimulation with LPS (100 ng/ml) for 3–6 hours. Cell lysates were prepared by washing cells in PBS prior to lysis in RIPA buffer (Tris 50 mM; NaCl 150 mM; SDS 0.1%; Na.Deoxycholate 0.5%; Triton X 100) containing phosphatase inhibitor cocktail set (Sigma-Aldrich). Samples were electrophoresed and transferred to PVDF prior to incubation with monoclonal antibodies specific for HO-1 (Enzo Life Sciences), pro-IL-1β, phospho-MEK, MEK, phospho-ERK, ERK, phospho-p38, p38 and IκB (all Cell Signaling), overnight at 4 °C. Membranes were then washed in TBS-Tween and incubated with appropriate streptavidin-conjugated secondary antibody (anti-rabbit, anti-mouse or anti-goat; all Sigma Aldrich) for 2 hours at room temperature, prior to development with enhanced chemiluminescent substrate (Merck Millipore) using a BioRad ChemiDoc MP system. Subsequently, membranes were re-probed with HRP-conjugated monoclonal antibodies specific for β-actin (Sigma-Aldrich) as a loading control.

### Confocal microscopy

Translocation of the active subunit of NFκB, p65, from the cytosol to the nucleus during LPS activation of DC was imaged by confocal microscopy. DC were seeded at 1 × 10^6^ cells/ml into a light-protective µ-Plate (iBidi) and treated with either carnosol (10 µM), curcumin (10 µM) or a vehicle control (DMSO) for 6 hours. DC were then left untreated, or stimulated with LPS (100 ng/ml). After 30 minutes, the µ-Plate was spun at 110 g for 5 minutes and cells were fixed with 4% paraformaldehyde in PBS for 15 minutes at room temperature. Cells were permeabilised by incubation in 100% methanol for 5 minutes at −20 °C. Following fixation and permeabilisation, cells were incubated with blocking buffer (5% BSA, 0.005% Tween-20 in PBS) for 1 hour at room temperature. After blocking, cells were incubated with anti-p65 antibody in blocking buffer (1:300, Santa Cruz Biotechnology) for 3 hours at room temperature, followed by incubation with a fluorochrome-conjugated secondary anti-mouse antibody in blocking buffer (1:500, Abcam) for 1 hour at room temperature. Finally, nuclei were stained with 4′,6-diamidino-2-phenylindole (DAPI) in PBS (1:2000) for 5 minutes at room temperature, and washed in PBS. Images were taken on a Leica SP8 scanning confocal microscope at X63 magnification.

### DC flow cytometry experiments

DC were collected, washed in PBS and stained extracellularly with amine-binding markers for dead cells (Fixable Viability Dye; eBioscience) and fluorochrome-conjugated antibodies for CD40, CD80, CD83, and CD86 (all eBioscience). For viability assays, DC were stained using an Annexin V & PI staining kit (eBioscience) to manufacturer’s instructions. For phagocytosis assays, DC were cultured with complete RPMI containing DQ-Ovalbumin (500 ng/ml; Invitrogen) for 20 minutes at 37 °C, followed by incubation for 10 minutes at 4 °C. DC were then washed in PBS and immediately acquired. Acquisition was performed on either a BD FACS Canto II or LSR Fortessa, and analysis was performed with FlowJo v.10 software (Tree Star Inc.). Gating strategies utilised in all experiments are detailed in Supplemental Fig. 10.

### Psoriasis PBMC flow cytometry

PBMC were re-stimulated in IMDM medium supplemented with 10% FCS, 2 mM L-glutamine, 100 U/ml penicillin and 100 µg/ml streptomycin (all Sigma Aldrich), and in the presence of 50 ng/ml phorbol 12-myristate 13-acetate (PMA), 500 ng/ml ionomycin and 5 µg/ml brefeldin A (all Sigma Aldrich) for 6 hours. Cells were then washed in PBS before viability staining (Fixable Viability Dye, eBioscience). Cells were then washed in PBS and surface stained with fluorochrome-conjugated antibodies targeting CD3, CD8, T-cell receptor γδ, and CD161 (BD Biosciences), for 20 minutes at room temperature. Cells were then fixed and permeabilized (FoxP3 Staining Buffer Set, eBioscience) for 30 minutes at 4 °C. Cells were then washed in permeabilization buffer and stained for intracellular Ki67, IL-2, IL-17A, IFNγ, (BD Biosciences), GM-CSF, TNFα and IL-22 (eBioscience) for 30 minutes at 4 °C. Samples were then washed in PBS and acquired on a BD LSRFortessa flow cytometer within 24 hours. Analysis was performed with FlowJo v.10 (Tree Star Inc.) and SPICE software (National Institute of Allergy and Infectious Diseases, National Institutes of Health). The gating strategy utilised is detailed in Supplemental Fig. 11.

### Statistical analysis

Statistical analysis was performed using Prism 6 software (GraphPad Software Inc.). Analysis of 3 or more data sets was performed by one-way ANOVA with either Tukey’s, Dunnett’s or Sidak’s post hoc test as appropriate; p values < 0.05 were considered significant and are denoted with asterisks or hash marks in the figures.

## Results

### Carnosol and curcumin are non-toxic and upregulate HO-1 expression in human DC

**Figure 1:**
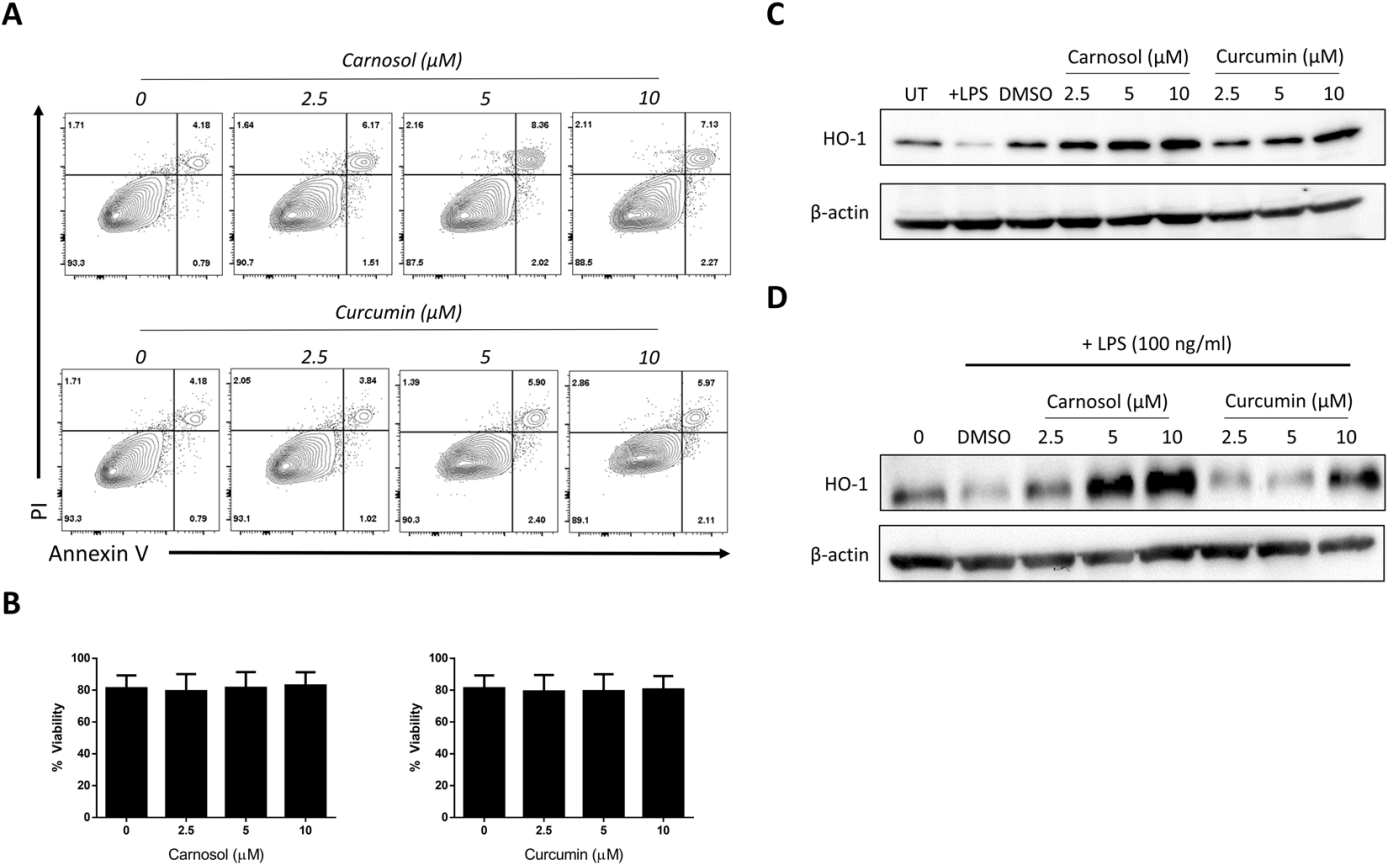
Carnosol and curcumin are non-toxic and induce HO-1 expression in human DC. (**A**) Primary human DC were incubated with a vehicle control, carnosol or curcumin (2.5–10 µM). After 24 hours, cells were stained for Annexin V and PI uptake and analysed by flow cytometry. Viable cells were designated as Annexin V^−^ PI^−^ (non-apoptotic cells). Results shown are from one healthy donor and are representative of data from four donors. (**B**) DC from healthy donors (n = 6) were incubated with increasing doses of carnosol (2.5–10 µM) or curcumin (2.5–10 µM) for 6 hours prior to stimulation with LPS (100 ng/ml). Cells were stained with a fixable viability dye after 24 hours and analysed by flow cytometry. Pooled data (n = 6) depicting the mean (±SEM) percentage viable cells of DC (gated by scatter, see Supplemental Fig. 10) (**C**) Immature DC were treated with vehicle control, LPS (100 ng/ml), carnosol or curcumin (2.5–10 µM) for 24 hours or (**D**) treated with carnosol or curcumin for 6 hours prior to stimulation with LPS for 24 hours. HO-1 expression was detected by western blot. The blots shown are derived from the same gel; membranes were first probed for HO-1 and then re-probed for β-actin. Full length blots are presented in Supplemental Fig. 2.

In order to confirm that carnosol and curcumin are non-toxic *in vitro* and capable of upregulating HO-1 in human DC at the concentrations used in this study, DC were treated with carnosol or curcumin (2.5–10 µM) and examined for cell viability and HO-1 expression. Viability for carnosol- and curcumin-treated DC was comparable to control-treated DC at all concentrations of carnosol and curcumin tested (Fig. 1A,B). HO-1 expression was assessed in carnosol- and curcumin-treated DC by western blot. Immature DC constitutively expressed HO-1 (Fig. 1C, lane 1), and consistent with previous reports^17–19^, LPS-stimulated mature DC downregulated HO-1 expression (Fig. 1C, lane 2). Conversely, treatment with carnosol or curcumin alone enhanced HO-1 expression (Fig. 1C, lanes 4–9). We also assessed the effect of carnosol and curcumin on HO-1 expression in the presence of LPS. As before, the basal expression of HO-1 was reduced in LPS-stimulated DC (Fig. 1D, lane 2), however this was overcome in the presence of carnosol or curcumin (Fig. 1D, lanes 3–8).

### Treatment with carnosol or curcumin inhibits LPS-mediated maturation of human DC

**Figure 2:**
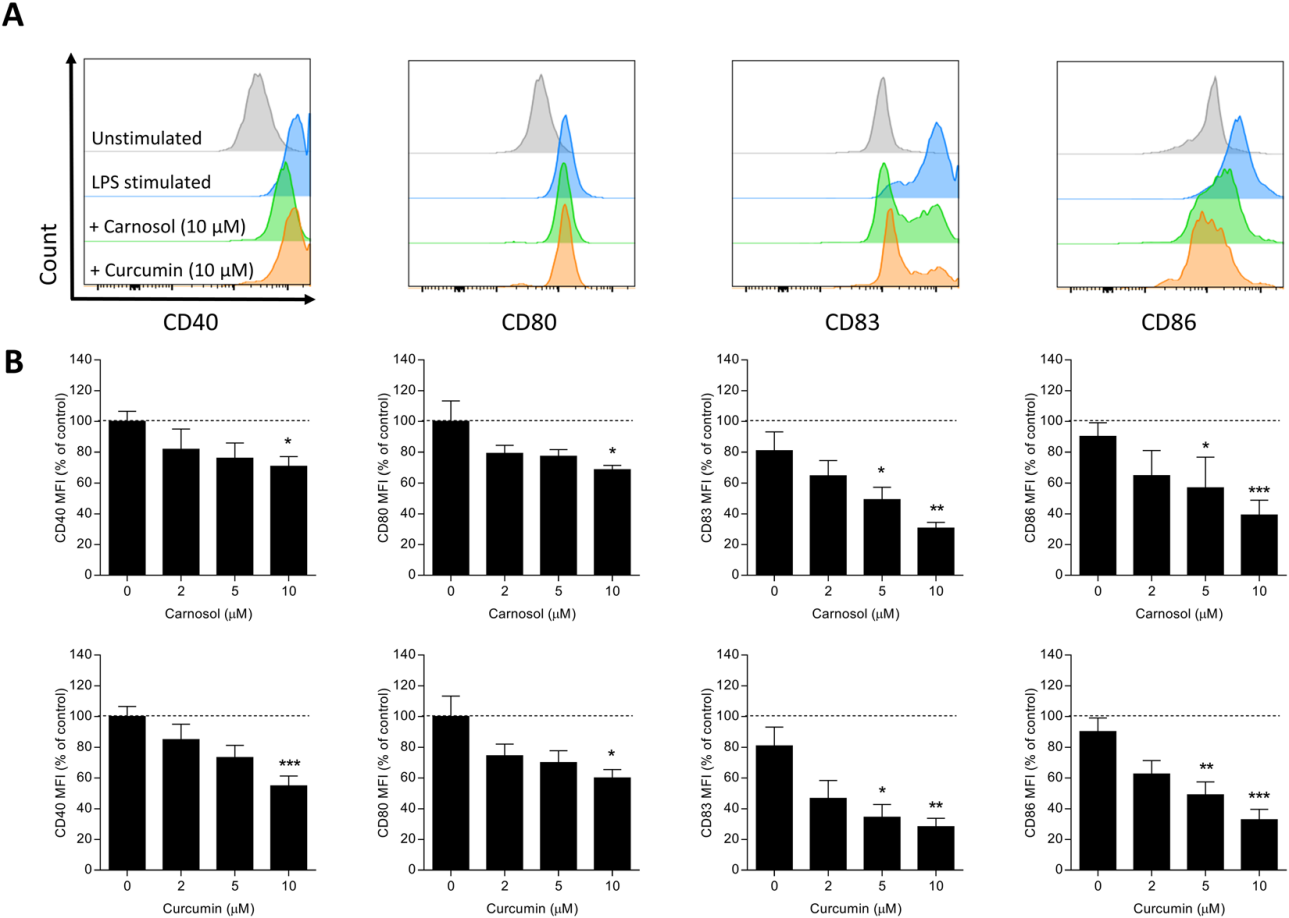
Treatment of LPS-stimulated DC with carnosol or curcumin down-modulates expression of CD40, CD80, CD83 and CD86. DC from healthy donors (n = 6) were incubated with increasing doses of carnosol (2.5–10 µM) or curcumin (2.5–10 µM) for 6 hours prior to stimulation with LPS (100 ng/ml). Cells were stained with fluorochrome conjugated antibodies specific for CD40, CD80, CD83 and CD86 after 24 hours and analysed by flow cytometry. (**A**) Histograms depicting expression of maturation markers in carnosol and curcumin treated DC compared to vehicle control from one representative experiment. (**B**) Pooled data (n = 6) depicting expression of CD40, CD80, CD83 and CD86 in carnosol and curcumin treated DC. Results shown are mean (±SEM) of the measured Mean Fluorescence Intensities (MFI), expressed as percentages of the vehicle controls. Statistical significance was determined by one-way ANOVA, with Dunnett’s multiple comparisons post hoc test to compare treatment groups against the control group. (***p < 0.001, **p < 0.01, *p < 0.05).

Mature DC are capable of activating naïve T cells and initiating abnormal immune responses which contribute to psoriasis pathology^3^. HO-1 overexpression with the metalloporphyrin cobalt protoporphyrin IX (CoPP) has previously been shown to arrest the maturation of DC via inhibition of surface co-stimulatory and maturation marker upregulation^18,35,36^. Similar observations have been reported in curcumin-treated DC in response to LPS stimulation^29,37^. Having confirmed that both carnosol and curcumin upregulate HO-1 expression in human DC, we next investigated whether they could also inhibit DC maturation in response to a pro-inflammatory stimulus such as LPS. Immature DC were incubated with increasing concentrations of carnosol or curcumin for 6 hours prior to stimulation with LPS. Expression of the co-stimulatory molecules CD80 and CD86, and the maturation markers CD40 and CD83 was measured by flow cytometry. Both carnosol and curcumin significantly downregulated expression of all markers tested compared to control DC, and these effects were observed to be dose-dependent (Fig. 2).

### Carnosol and curcumin treatment maintains the phagocytic capacity of LPS-stimulated DC in a HO-1-dependent manner

**Figure 3:**
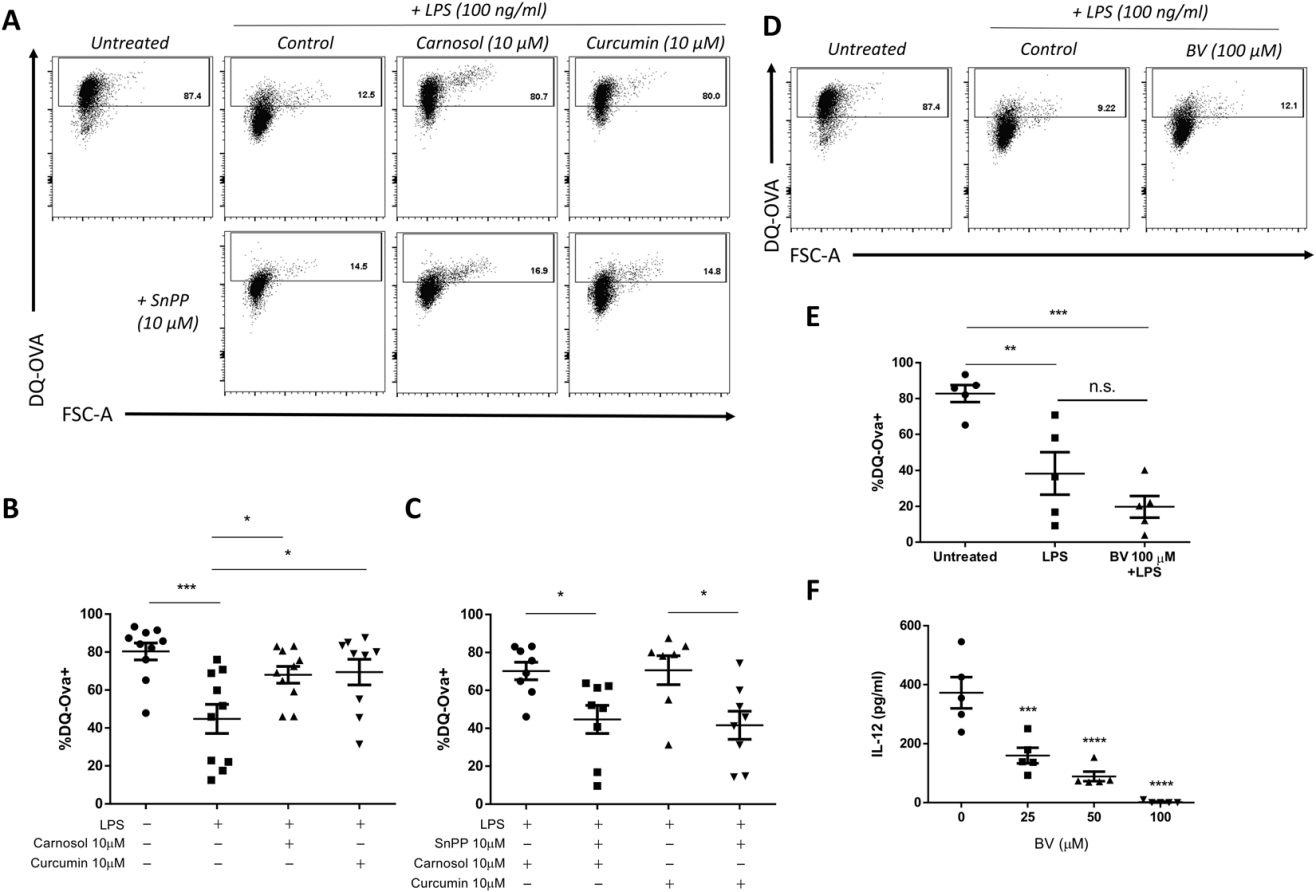
Carnosol and curcumin maintenance of antigen uptake in LPS-treated DC is dependent on HO-1 but not biliverdin. DC from healthy donors (n = 8–10) were incubated with carnosol (10 µM) or curcumin (10 µM) with or without SnPP (10 µM) for 6 hours prior to stimulation with LPS for 24 hours. DC were then incubated with DQ-Ovalbumin (DQ-Ova; 500 ng/ml) for 20 minutes prior to analysis by flow cytometry. (**A**) Representative dot plots depicting DQ-Ova uptake by DC treated with carnosol, curcumin and SnPP are from one donor. (**B**) Pooled data depicting percentage DQ-Ova uptake of DC treated with carnosol and curcumin alone and (**C**) in the presence of SnPP. (**D**) DC from healthy donors (n = 5) were incubated with biliverdin (BV; 100 µM) for 6 hours prior to stimulation with LPS for 24 hours. DC were then incubated with DQ-Ova and analysed for antigen uptake as before. (**E**) Pooled data depicting mean (±SEM) percentage DQ-Ova uptake of DC treated with BV. (**F**) Pooled data depicting mean (±SEM) concentration of IL-12 in supernatants of BV treated DC as measured by ELISA (mean of three technical replicates per donor). Statistical significance was determined by one-way ANOVA, with either Tukey’s multiple comparisons post hoc test to compare means of all groups, or Sidak’s multiple comparisons post hoc test to compare means of pre-selected group pairs. (****p < 0.0001, ***p < 0.001, **p < 0.01, *p < 0.05).

Upon maturation, DC lose their phagocytic ability as their role switches from antigen uptake to antigen presentation^38,39^. As we had previously observed that carnosol and curcumin reduced phenotypic maturation of DC, we next investigated whether carnosol or curcumin treatment might also maintain the phagocytic capacity of DC after LPS stimulation. To test this, immature DC were pre-treated with carnosol (10 µM) or curcumin (10 µM) for 6 hours prior to stimulation with LPS. After 24 hours, DC were incubated with FITC-conjugated DQ-Ovalbumin (DQ-Ova) at 500 ng/ml and analysed for antigen uptake by flow cytometry. As expected, immature DC displayed high DQ-Ova uptake, and this was significantly reduced in LPS-stimulated DC. However, both carnosol and curcumin treatment maintained the phagocytic capacity of LPS-treated DC, with DQ-Ova uptake levels observed to be similar to that of immature DC (Fig. 3A,B). In order to determine if this effect is dependent on HO-1 activity, immature DC were treated with carnosol (10 µM) or curcumin (10 µM) in the presence or absence of SnPP (10 µM), a competitive HO-1 enzymatic inhibitor, prior to LPS stimulation and DQ-Ova treatment as before. Our results show that the increase in DQ-Ova uptake in carnosol- and curcumin-treated DC stimulated with LPS was significantly attenuated in the presence of SnPP (Fig. 3A,C).

We have previously reported that the HO-1 reaction product, biliverdin, reduces DC maturation marker expression and pro-inflammatory cytokine production^40^. We therefore hypothesised that biliverdin may be mediating the effect of carnosol/curcumin-induced HO-1. However, in contrast to carnosol or curcumin, biliverdin-treated DC stimulated with LPS did not show any increase in antigen uptake capacity compared to control DC (Fig. 3D,E). Biliverdin treatment did, however, significantly inhibit IL-12p70 production (Fig. 3F) suggesting that HO-1-produced CO, rather than biliverdin, may play a more prominent role in maintaining phagocytic capacity.

### Carnosol and curcumin reduce pro-inflammatory cytokine production by LPS-stimulated human DC

**Figure 4:**
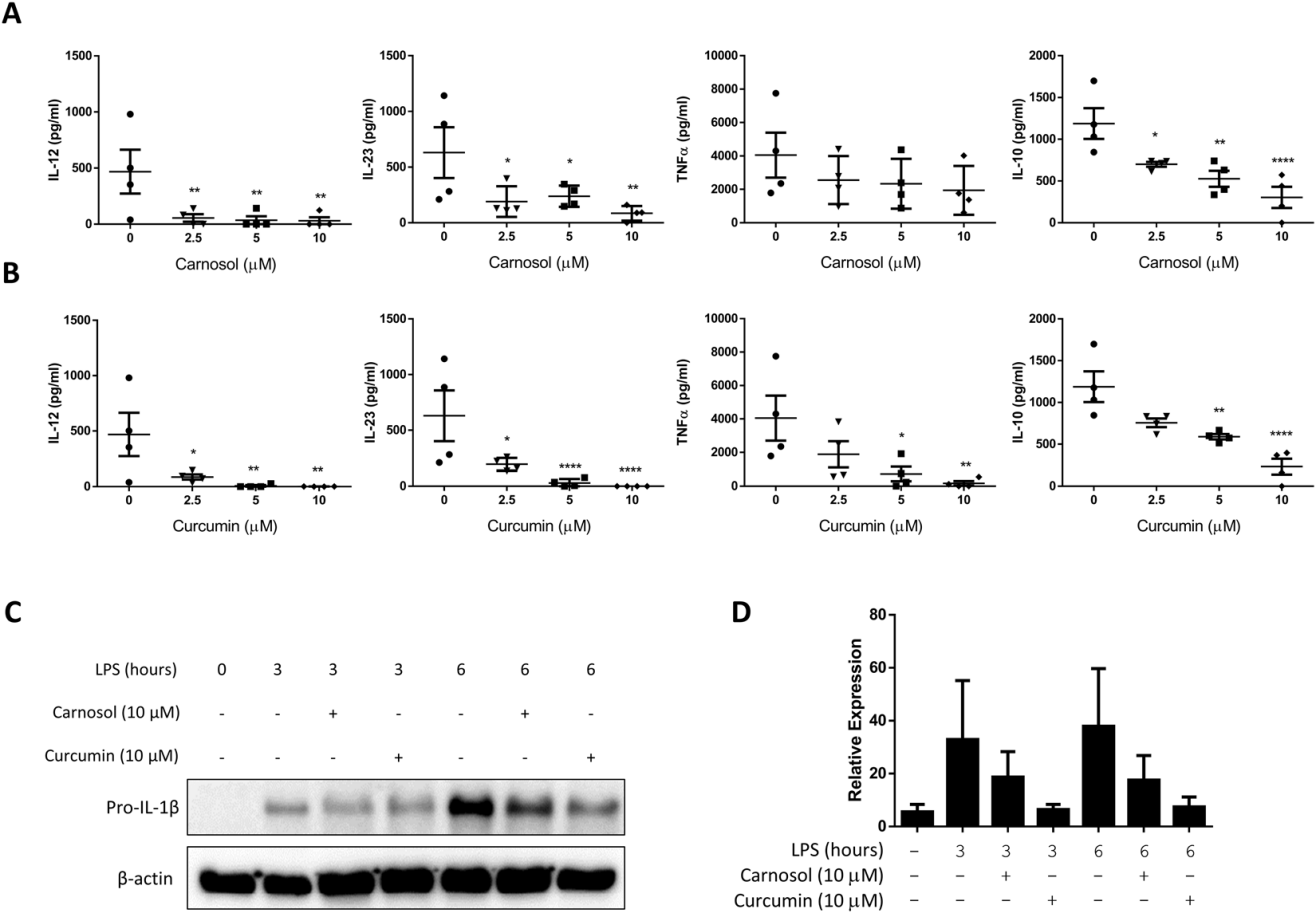
Carnosol and curcumin inhibit cytokine production in LPS-treated DC. DC from healthy donors (n = 4) were incubated with increasing doses of (**A**) carnosol (2.5–10 µM) or (**B**) curcumin (2.5–10 µM) for 6 hours prior to stimulation with LPS. Supernatants were collected after 24 hours and IL-12p70, IL-23p19, TNFα, and IL-10 production was measured by ELISA. Results shown are mean (±SEM) of the pooled cytokine concentrations (means of three technical replicates per donor). Statistical significance was determined by one-way ANOVA, with Dunnett’s multiple comparisons post hoc test to compare means of treatment groups to the control group. (****p < 0.0001, ***p < 0.001, ** p < 0.01, *p < 0.05). (**C**) DC were treated with either carnosol (10 µM), curcumin (10 µM) or a vehicle control for 6 hours prior to stimulation with LPS. Cell lysates were harvested at 3 hours and 6 hours post LPS stimulation and expression of pro-IL-1β was detected by western blotting. Representative blot of three independent experiments is shown. The blots shown are derived from the same gel; membranes were first probed for pro-IL-1β and then re-probed for β-actin. Full length blots are presented in Supplemental Fig. 3. (**D**) Densitometric analysis of 3 immunoblots was performed using ImageLab (Bio-Rad) software.

Cytokine production by DC plays a significant role in the pathology of psoriasis: TNFα contributes to inflammation in the skin, while IL-12 and IL-23 instruct T cell polarisation towards the pro-inflammatory Th1 and Th17 phenotypes, respectively^41–43^. To examine the effect of carnosol and curcumin on cytokine production by human DC, immature DC were pre-treated with carnosol or curcumin and stimulated with LPS for 24 hours as before. Supernatants were collected and analysed by ELISA for IL-12p70, IL-23p19, TNFα and IL-10. Both carnosol and curcumin treatment reduced IL-12p70 and IL-23p19 to almost undetectable levels, even at the lowest concentrations of carnosol and curcumin tested. Curcumin also significantly decreased the concentration of TNFα, while there was a non-significant trend towards reduced TNFα in carnosol-treated DC. A decrease in the anti-inflammatory cytokine, IL-10, was also observed with both carnosol and curcumin treatment however the ratio of IL-10 to pro-inflammatory cytokines remained favourable (Fig. 4A,B). IL-1β plays a crucial role in innate immune responses and is also associated with Th17 differentiation^44^. Unlike the cytokines listed above, IL-1β is not secreted into DC cell supernatants by LPS stimulation alone as the immature cytokine (pro-IL-1β) must be processed by caspase-1 and the inflammasome complex prior to secretion. It is, however, possible to detect the intracellular pro-form of IL-1β by western blot. To examine production of pro-IL-1β, immature DC were pre-treated with carnosol (10 µM) or curcumin (10 µM) for 6 hours prior to stimulation with LPS for 3–6 hours. Pro-IL-1β expression was upregulated in whole cell lysates from DC treated with LPS in comparison to unstimulated DC, however this effect was reduced in LPS-treated DC that had been pre-treated with either carnosol or curcumin (Fig. 4C,D).

### Carnosol and curcumin inhibit MAP Kinase activation in LPS-stimulated human DC

**Figure 5:**
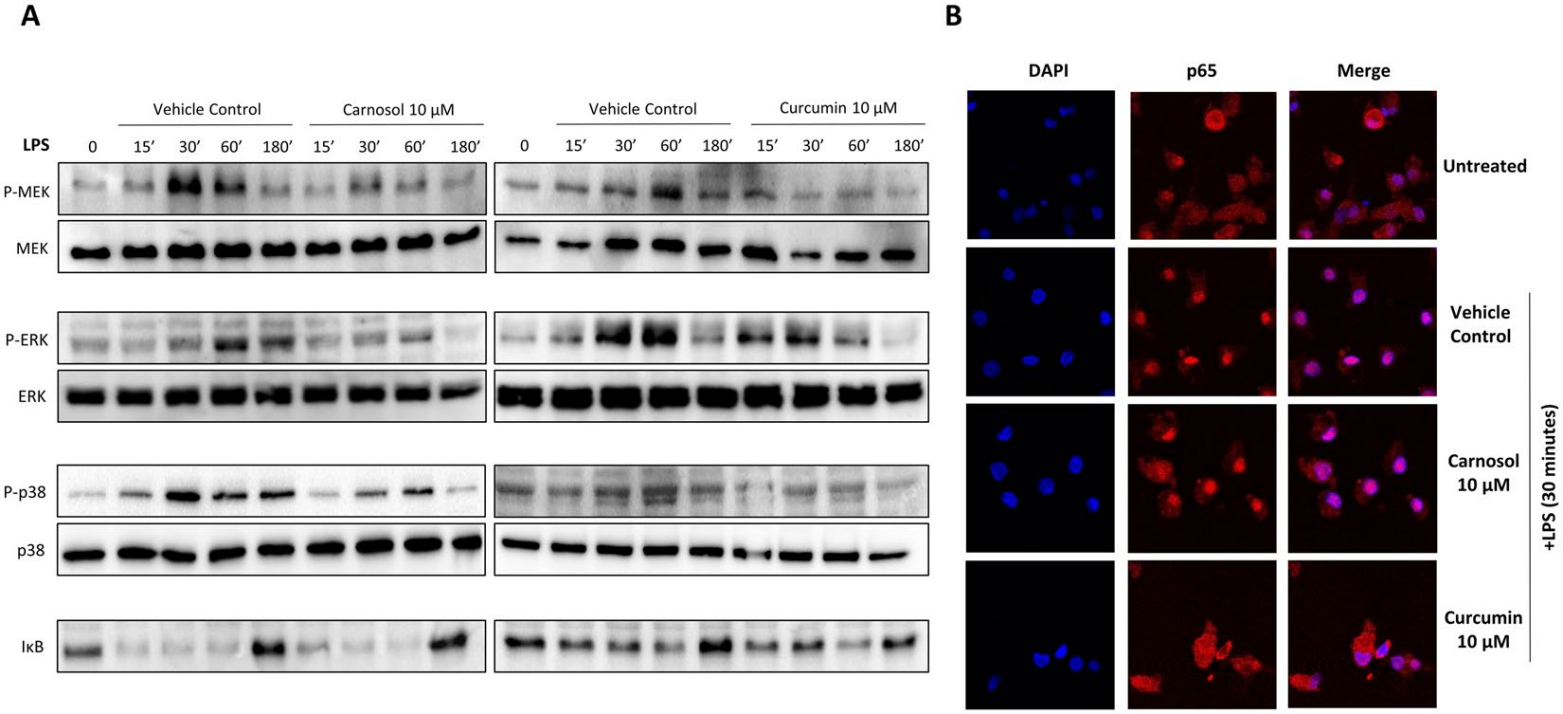
Carnosol and curcumin reduce activation of MAP Kinases, and curcumin reduces nuclear translocation of the NFκB subunit p65, in LPS-stimulated DC. DC from healthy donors (n = 6) were incubated with carnosol (10 µM), curcumin (10 µM) or a vehicle control for 6 hours prior to stimulation with LPS for 15 minutes to 3 hours. (**A**) The activation of the MAPKs MEK, ERK and p38, as well as degradation of IκB was measured by western blot. Data shown is representative of 6 healthy donors. Full length blots are presented in Supplemental Figs 4 and 5 (**B**) The nuclear translocation of the NFκB subunit p65 in vehicle control, carnosol or curcumin treated DC after stimulation with LPS for 30 minutes was assessed by confocal microscopy. Data shown is from one healthy donor and is representative of 4 healthy donors. Cropped images are presented for clarity; full size images are presented in Supplemental Fig. 6.

Signalling through MAPKs such as MEK, ERK and p38 is involved in the mediation of the functional and phenotypic changes arising from the maturation of human DC in response to pro-inflammatory stimuli^45^. Having observed that treatment with carnosol or curcumin inhibits the maturation and function of DC, experiments were performed to investigate whether inhibition of signalling via MEK, ERK or p38 could be a possible mechanism for these effects. DC were pre-treated with carnosol (10 µM) or curcumin (10 µM) for 6 hours prior to stimulation with LPS over time. Phosphorylation, and therefore, activation of MEK, ERK and p38 MAPK was assessed by western blotting. Both carnosol and curcumin treatment reduced LPS induced activation of MEK and ERK, while a modest reduction of p38 activation was also observed (Fig. 5A). Neither carnosol nor curcumin had any effect of the degradation of the NFκB inhibitor, IκB following LPS stimulation.

To ensure the observed reduction of MAPK activation was not due to early activation of MAPKs by carnosol and curcumin and therefore tolerance to LPS stimulation, we assessed activation of MEK (which acts upstream of ERK) and p38 over time after addition of carnosol, curcumin or LPS as a positive control. As expected, LPS induced activation of both MEK and p38, however there was no observed effect with either carnosol or curcumin (Supplemental Fig. 1). We also assessed translocation of the active NFκB subunit, p65, to the nucleus post LPS stimulation and again carnosol had no effect on p65 activation. Interestingly, LPS-induced translocation of p65 was inhibited with curcumin treatment suggesting that the two compounds may differentially affect NFκB (Fig. 5B).

**Figure 6:**
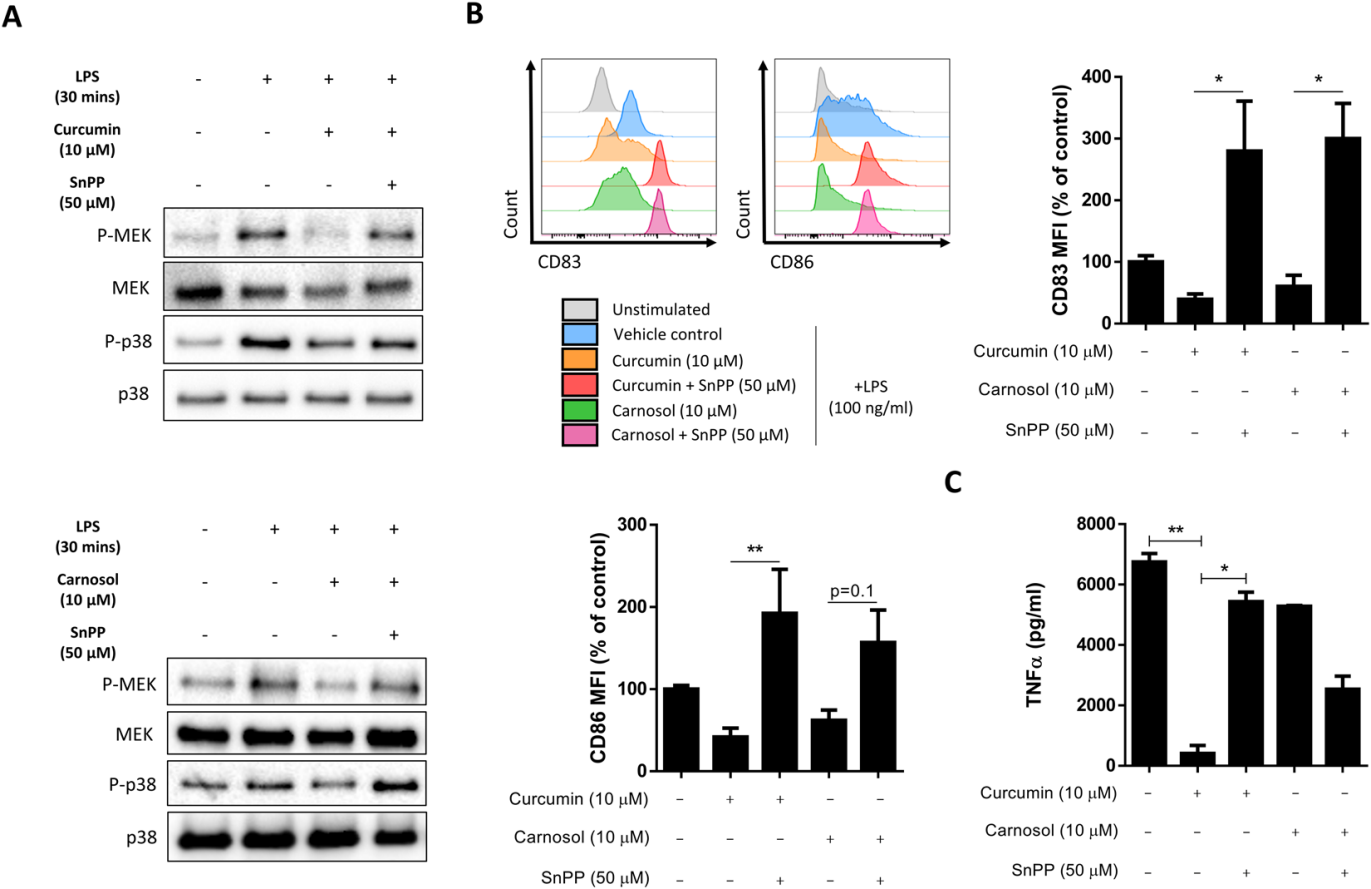
SnPP, a HO-1 inhibitor, attenuates the effects of carnosol and curcumin on MAP Kinase activation, DC maturation and pro-inflammatory cytokine production. DC from healthy donors (n = 3) were incubated with carnosol (10 µM) or curcumin (10 µM), in the presence or absence of SnPP (50 µM), for 6 hours prior to stimulation with LPS for 30 minutes. (**A**) The activation of the MAPKs MEK and p38 was measured by western blot. Data shown is representative of 3 healthy donors. Full length blots are presented in Supplemental Fig. 7. DC from healthy donors (n = 4) were incubated with carnosol (10 µM), curcumin (10 µM) or a vehicle control, in the presence or absence of SnPP (50 µM) for 6 hours prior to stimulation with LPS for 24 hours. (**B**) The expression of maturation markers CD83 and CD86 was measured by flow cytometry. Histograms depict expression of CD83 and CD86 in carnosol and curcumin treated DC, with or without SnPP, compared to control DC from one representative experiment. Pooled data (n = 4) shown are mean (±SEM) of the measured MFIs, expressed as percentages of the vehicle control. **(C) **The concentration of TNFα in cell culture supernatants was measured by ELISA. Results shown are mean (±SD) of two replicates from one healthy donor, and is representative of four independent experiments. Statistical significance was determined by one-way ANOVA, with Sidak’s multiple comparisons post hoc test to compare the means of preselected pairs of groups (**p < 0.01, *p < 0.05).

Finally, in order to confirm that inhibition of MAPK activation by carnosol and curcumin is dependent on HO-1, DC were pre-treated with carnosol (10 µM) or curcumin (10 µM), in the presence or absence of the HO-1 inhibitor SnPP (50 µM), for 6 hours prior to stimulation with LPS for 30 minutes. Phosphorylation of MEK and p38 MAPK was assessed by western blotting. As before, both carnosol and curcumin inhibited LPS induced activation of both MAPKs; however, this effect was abrogated in the presence of SnPP (Fig. 6A). Furthermore, inclusion of the HO-1 inhibitor attenuated the ability of carnosol and curcumin to reduce DC expression of CD83 and CD86 (Fig. 6B). Finally, we assessed effects on pro-inflammatory cytokine production. As previously observed, curcumin potently reduced LPS-induced TNFα production (with carnosol having little effect), however inclusion of the HO-1 inhibitor reversed this inhibition (Fig. 6C).

### The carbon monoxide scavenger, hemoglobin, reduces the anti-inflammatory effects of carnosol and curcumin in human DC

**Figure 7:**
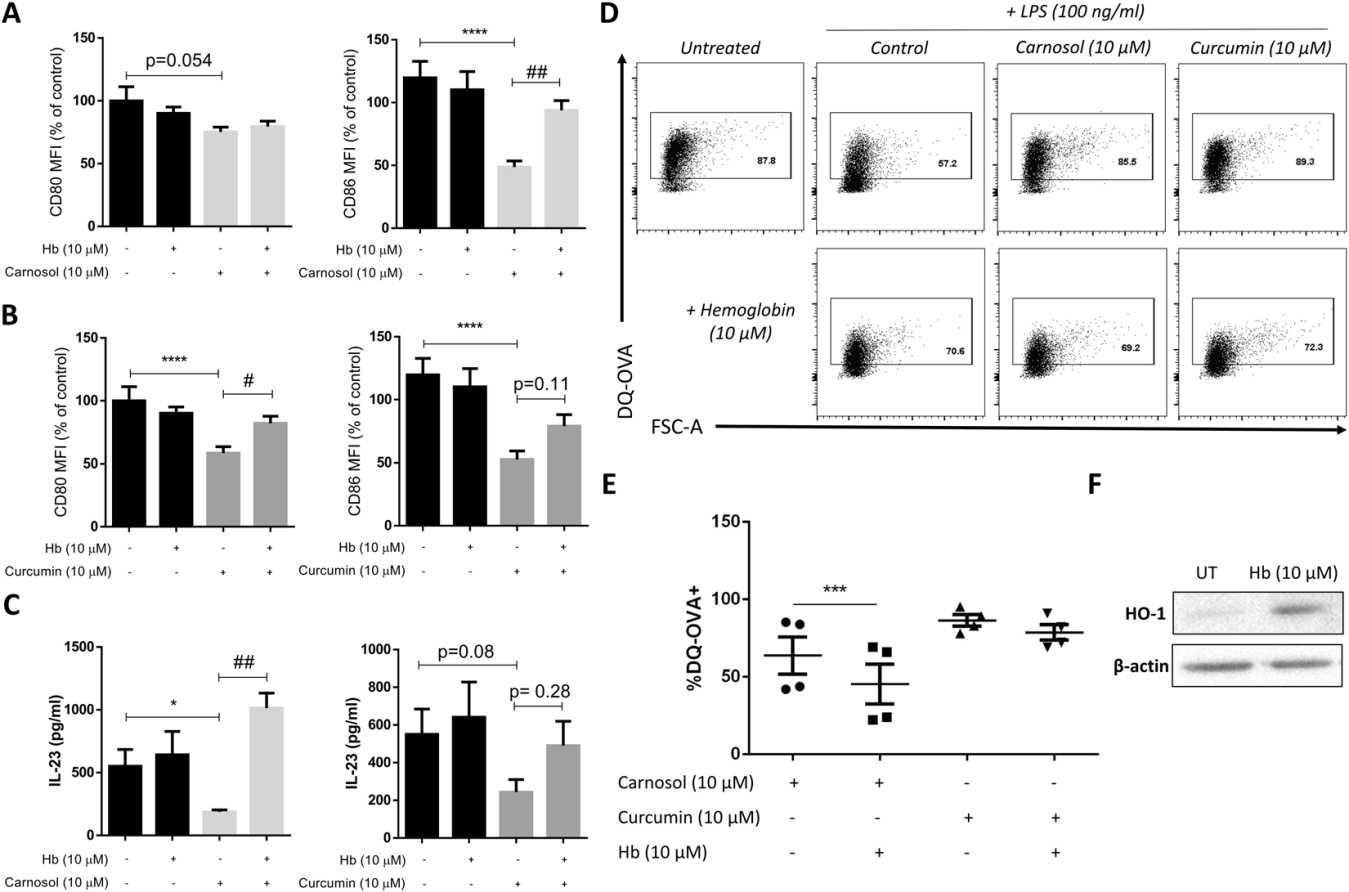
Hemoglobin, a CO scavenger, reduces the anti-inflammatory effects of carnosol and curcumin treatment in LPS-stimulated DC. DC from healthy donors (n = 3) were treated with carnosol (10 µM) or curcumin (10 µM) either alone or in the presence of the CO scavenger hemoglobin (Hb; 10 µM) for 6 hours prior to stimulation with LPS. Cells were stained with fluorochrome conjugated antibodies specific for CD80 and CD86 after 24 hours and analysed by flow cytometry. Pooled data depicting expression of CD80 and CD86 in (**A**) carnosol and (**B**) curcumin treated DC. Results shown are mean (±SEM) of the measured MFIs, expressed as percentages of the vehicle controls. (**C**) Pooled data depicting mean (±SEM) concentration of IL-23 in supernatants of DC treated with carnosol or curcumin, with or without the addition of Hb (means of three technical replicates per donor). (**D**) DC from healthy donors (n = 4) treated with carnosol (10 µM) or curcumin (10 µM), with or without Hb (10 µM), were incubated with DQ-OVA as described above. Representative dot plots depicting DQ-Ova uptake by DC treated with carnosol, curcumin and Hb are from one donor. (**E**) Pooled data depicting mean (±SEM) percentage DQ-Ova uptake of DC treated with carnosol and curcumin either alone or in the presence of Hb. (**F**) Western blot depicting HO-1 expression in DC either untreated (UT) or treated with Hb (10 µM) for 24 hours. Full length blot is presented in Supplemental Fig. 8. Statistical significance was determined by one-way ANOVA, with Dunnett’s multiple comparisons post hoc test to compare treatment groups against the control group (denoted by asterisks) and Sidak’s multiple comparisons post hoc test to compare the means of preselected pairs of groups (denoted by hash marks). (****p < 0.0001, *p < 0.05, ^###^p < 0.001, ^##^p < 0.01, ^#^p < 0.05).

The HO-1 reaction product CO has previously been demonstrated to inhibit DC maturation and antigen presentation to T cells^46–48^. To test whether CO generation contributes to the activity of carnosol and curcumin, DC were treated with carnosol and curcumin, as before, with the addition of hemoglobin (Hb), which strongly binds CO and has been used previously as a scavenger of CO *in vitro*^21^. Addition of Hb resulted in a reversal of the previously observed reduction in CD86 expression in LPS-stimulated DC pre-treated with carnosol (Fig. 7A). In curcumin-treated DC, Hb partially reversed reductions in both CD80 and CD86 expression after LPS stimulation (Fig. 7B). Additionally, carnosol-treated DC stimulated with LPS displayed significantly higher IL-23 production when cultured in the presence of Hb and a trend towards increased IL-23 production was observed in curcumin-treated DC (Fig. 7C). Similar to HO-1 inhibition with SnPP, Hb treatment significantly attenuated DQ-Ova uptake in carnosol treated DC suggesting that CO is at least partially responsible for maintaining DC in an immature state post HO-1 induction. Hb also modestly reduced curcumin mediated DQ-Ova uptake, however the effects were not as potent as those observed with carnosol (Fig. 7D,E). Western blotting revealed that Hb was inducing HO-1 expression to some extent over the 24 hour time period (Fig. 7F) which may be masking some of the effects observed.

### Carnosol and curcumin-treated DC have a reduced capacity to stimulate proliferation of allogeneic CD4^+^ T cells

**Figure 8:**
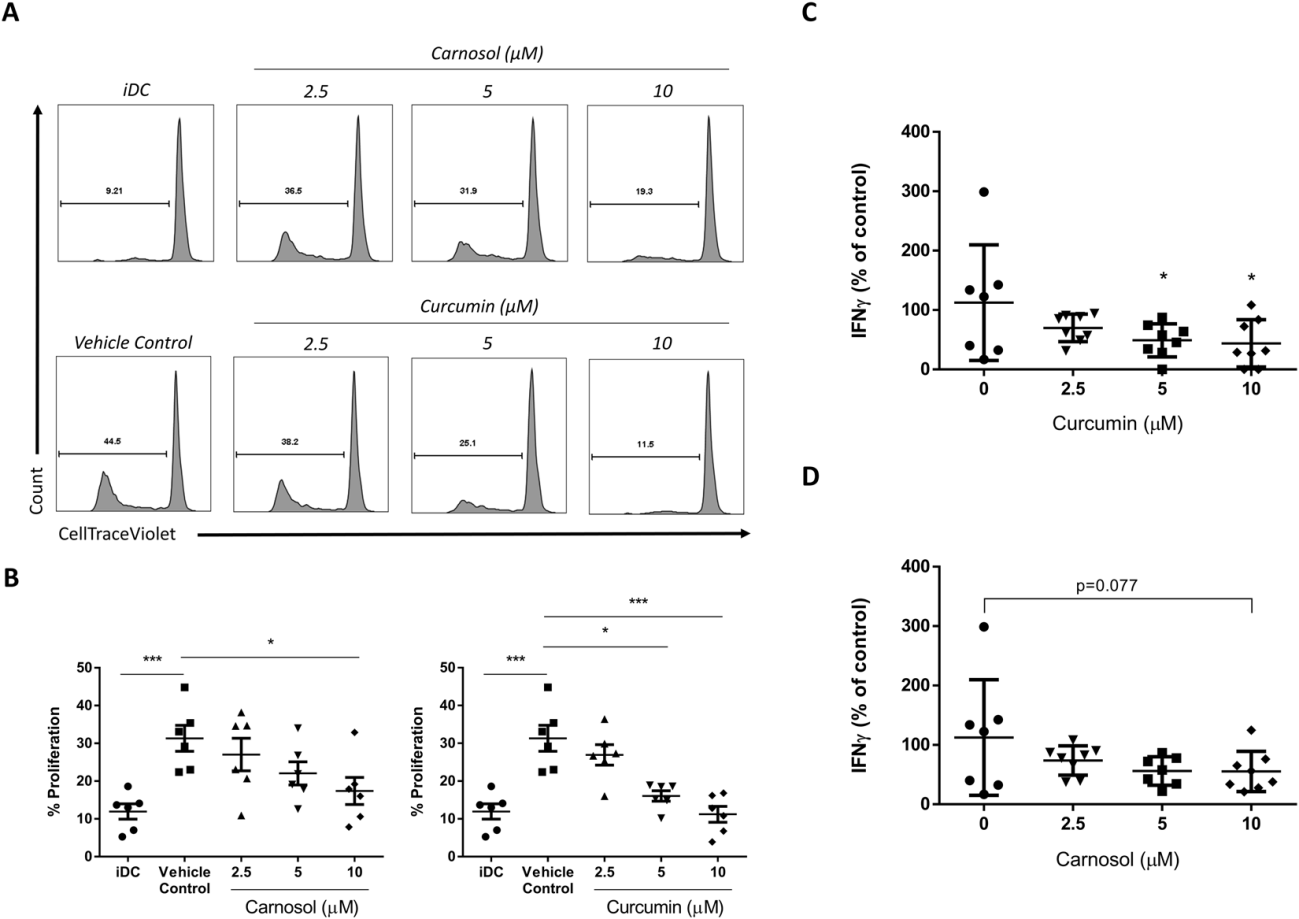
Carnosol and curcumin treated DC have a reduced capacity to stimulate allogeneic CD4^+^ T cells. DC from healthy donors (n = 6–7) were treated with carnosol or curcumin (2.5–10 µM) for 6 hours prior to stimulation with LPS. After 24 hours, DC were washed and cultured at a 1:10 ratio with allogeneic purified CD4^+^ T cells. Immature DC (iDC) were also cultured with CD4^+^ T cells as an allogeneic control. After 5 days CD4^+^ T cells were analysed for proliferation by flow cytometry and supernatants were measured for IFNγ concentration by ELISA. (**A**) Histograms depicting proliferation of CD4^+^ T cells co-cultured with either control, carnosol or curcumin treated allogeneic DC, as measured by CTV fluorescence. Data shown is from one healthy donor. (**B**) Pooled data depicting mean (±SEM) percentage proliferation of CD4^+^ T cells cultured with control DC or DC treated with different concentrations of carnosol or curcumin. Statistical significance was determined by one-way ANOVA, with Tukey’s multiple comparisons post hoc test to compare means of all groups. Pooled data depicting mean (±SEM) percentage reduction in IFNγ in T cells cultured with (**C**) curcumin or (**D**) carnosol treated DC matured with LPS. Statistical significance was determined by one-way ANOVA, with Dunnett’s multiple comparisons post hoc test to compare means of all treatment groups to the control group. (****p < 0.0001, ***p < 0.001, **p < 0.01, *p < 0.05).

The immune synapse between DC and CD4^+^ T cells is central to the generation of harmful Th1 and Th17 responses in psoriasis; thus, the overall goal in modulating DC maturation and function in psoriasis is to limit the activation of adaptive immune responses which drive psoriatic inflammation. It has previously been reported that HO-1-overexpressing DC have an impaired ability to drive adaptive T cell responses^18,19,37^. In order to assess the impact of carnosol and curcumin treatment on the ability of DC to activate T cells, immature DC were pre-treated with carnosol or curcumin prior to stimulation with LPS as previously described. After 24 hours, cells were washed in fresh RPMI and added to purified allogeneic CD4^+^ T cells stained with CTV. After 5 days, CD4^+^ T cell proliferation was measured by CTV fluorescence and supernatants were analysed for IFNγ production as an indication of T cell activation. Analysis of CD4^+^ T cell proliferation revealed that T cells cultured with allogeneic DC, previously matured with LPS, demonstrated a higher level of proliferation compared to T cells cultured with immature DC. However, CD4^+^ T cells cultured with LPS-stimulated DC, previously treated with carnosol or curcumin, showed significantly lower levels of proliferation, and this effect was observed to be dose-dependent (Fig. 8A,B). Similarly, the concentration of IFNγ in the supernatants of T cells cultured with carnosol- or curcumin-treated DC was significantly lower than that from T cells cultured with LPS-stimulated DC (Fig. 8C,D) suggesting that carnosol and curcumin reduce the capacity of DC to stimulate allogeneic T cells.

### Curcumin, but not carnosol, significantly reduced T cell proliferation and cytokine production in *ex-vivo* psoriasis PBMC

Having demonstrated that carnosol and curcumin are capable of attenuating DC maturation, pro-inflammatory cytokine production and generation of adaptive T cell responses by human DC, we next examined whether these polyphenols also have anti-inflammatory effects on PBMC from psoriasis patients. PBMC were isolated from the blood of psoriasis patients and treated with carnosol (5 µM), curcumin (5 µM) or vehicle control for 6 hours prior to stimulation with anti-CD3 to induce T cell activation. After 4 days PBMC were re-stimulated with PMA and ionomycin in the presence of brefeldin A to assess T cell cytokine production and proliferation by flow cytometry. The gating strategy used for analysis is described in Supplemental Fig. 11; briefly, lymphocytes were gated based on forward and side scatter, followed by single cells and live cells as determined by viability-dye exclusion. Within the live cell gate, both CD3^+^TCRγδ^+^ and CD3^+^CD8^−^ cell populations were gated on; analysis of proliferation and cytokine production was performed within the CD3^+^CD8^−^ population (comprised mostly of CD4^+^ T cells and a small percentage of γδ T cells).

**Figure 9:**
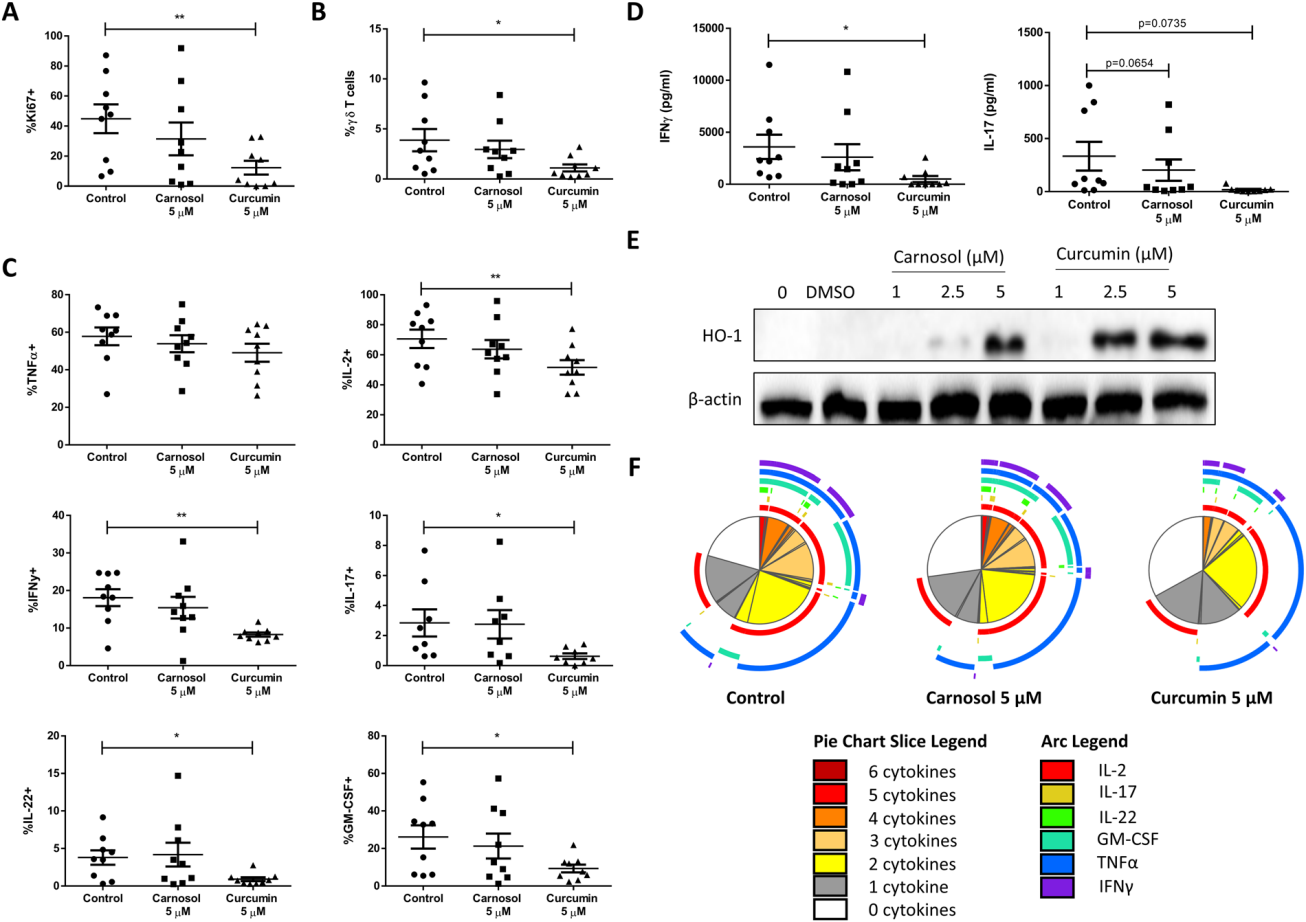
Curcumin, but not carnosol, significantly inhibits T cell immune responses in *ex-vivo* stimulated PBMC from psoriasis patients. PBMC isolated from psoriasis patients (n = 9) were treated with carnosol (5 µM), curcumin (5 µM) or a vehicle control for 6 hours prior to stimulation with anti-CD3. After 4 days, supernatants were removed for analysis of IFNγ and IL-17 concentration by ELISA, and proliferation and cytokine production by T cells was analysed by flow cytometry. CD3^+^CD8^-^ T cell populations were gated according to the strategy shown in Supplemental Fig. 11. (**A**) Pooled data depicting mean (±SEM) percentage expression of the proliferation marker Ki67 in CD3^+^CD8^-^ T cells from control, carnosol or curcumin treated PBMC. (**B**) Pooled data depicting mean (±SEM) CD3^+^TCRγδ^+^ cells as a percentage of total live cells from control, carnosol or curcumin treated PBMC. (**C**) Pooled data depicting mean (±SEM) percentages of TNFα, IL-2, IFNγ, IL-17A, GM-CSF and IL-22 positive CD3^+^CD8^−^ T cells from control, carnosol or curcumin treated PBMC. (**D**) Pooled data depicting mean (±SEM) concentrations of IFNγ and IL-17 in cell culture supernatants of control, carnosol and curcumin treated PBMC (means of three technical replicates per patient). (**E**) PBMC from a healthy human donor were treated with carnosol (1–5 µM), curcumin (1–5 µM), or a vehicle control for 24 hours. Expression of HO-1 was measured by western blot. The blots shown are derived from the same gel; membranes were first probed for HO-1 and then re-probed for β-actin. Full length blots are presented in Supplemental Fig. 9. (**F**) The cytokine expression profiles of T cells from control, carnosol and curcumin treated psoriasis PBMC were analysed using SPICE software. The pie charts represent the average frequencies of CD3^+^CD8^-^ T cells producing every combination of cytokines for multiple patients (n = 9). The segments within the pie charts denote proportions of the CD3^+^CD8^−^ T cell population producing different combinations of cytokines, and are heat-map coded to indicate increasing cytokine production. The arcs surrounding the pie charts indicate the cytokine(s) produced by that proportion of cells. Statistical significance was determined by one-way ANOVA with Dunnett’s post hoc test to compare carnosol and curcumin treatment to the control (**p < 0.01, *p < 0.05).

Proliferation of CD3^+^CD8^−^ T cells, as measured by expression of the nuclear proliferation marker Ki67, was significantly reduced in curcumin, but not carnosol, treated psoriasis PBMC (Fig. 9A). Recently, an important role for γδ T cells in psoriasis pathogenesis has been described in murine models^49,50^. Whether they are a significant T cell population in human disease remains unclear, however, we found that curcumin effectively limited the *ex-vivo* expansion of γδ T cells from psoriasis PBMC (Fig. 9B). The expression of cytokines which characterise different Th cell subtypes and are relevant mediators of the T cell immune response in psoriasis was analysed in CD3^+^CD8^−^ T cells from psoriasis PBMC. As expected, the majority of CD3^+^CD8^−^ T cells produced TNFα and IL-2; there was no reduction in TNFα with either carnosol or curcumin treatment, and only a small reduction in IL-2 production was seen in curcumin treated psoriasis PBMC. In contrast, curcumin treatment resulted in greater than 50% reductions in the production of pro-inflammatory cytokines including the Th1 cytokine IFNγ and Th17-associated cytokines IL-17, GM-CSF and IL-22. There were no significant differences observed in the frequencies of any cytokine-producing CD3^+^CD8^−^ T cells between carnosol and control treated psoriasis PBMC (Fig. 9C).

The effects of carnosol and curcumin on pro-inflammatory cytokine production by psoriasis PBMC was confirmed by analysis of IFNγ and IL-17 concentrations in cell culture supernatants by ELISA. A significant reduction in IFNγ concentration was observed with curcumin but not carnosol treatment, while a trend towards reduced IL-17 concentrations was observed in both carnosol and curcumin treated psoriasis PBMC (Fig. 9D). Due to limited cell numbers it was not possible to measure HO-1 induction by carnosol and curcumin in psoriasis PBMC, however HO-1 expression was confirmed in healthy human PBMC upon treatment with carnosol or curcumin (1–5 µM). Unlike human DC, HO-1 is not basally expressed in PBMC, however, upregulation of HO-1 was observed in both carnosol and curcumin treated PBMC at 2.5 and 5 µM, confirming that they are capable of inducing the enzyme at the concentrations used in this study (Fig. 9E).

### Reduction in cytokine poly-functionality in curcumin-treated psoriasis PBMC

Given that one of the hallmarks of highly pro-inflammatory T cells is the capacity to produce multiple different cytokines, we next used SPICE software (National Institute of Allergy and Infectious Diseases, National Institute of Health, Bethesda, MD) to analyse the poly-functionality of CD3^+^CD8^−^ T cells in control, carnosol and curcumin treated psoriasis PBMC (Fig. 9F). The segments within the pie charts represent the proportion of cells producing 0 to 6 cytokines simultaneously, and are heat-map coded to indicate increasing cytokine poly-functionality (pie chart legend: white to red). The size of a pie segment corresponds to the frequency of that population. The arcs surrounding the pie charts correspond to the specific cytokines produced by the pie segments underneath (see pie arc legend). By comparing the poly-functionality profiles of control-treated to carnosol and curcumin treated psoriasis PBMC it was observed that there was no difference in the frequencies of poly-functional CD3^+^CD8^−^ T cells in carnosol treated psoriasis PBMC compared to control. In contrast, a clear increase in the frequency of T cells producing no cytokines (white pie segment) and decrease in the frequencies of T cells producing 3 or more cytokines (orange and red segments) was observed in curcumin treated psoriasis PBMC compared to control. By examining the cytokine arcs it is evident that this decrease in T cell poly-functionality in curcumin treated psoriasis PBMC corresponds with a reduction in the frequencies of IFNγ, IL-17, GM-CSF and IL-22 producing cells, as was seen in Fig. 9C.

## Discussion

Psoriasis is a prevalent, chronic autoimmune disease of the skin in which excessive inflammation causes epidermal hyperplasia, resulting in the formation of psoriatic plaques. Although the exact mechanisms of psoriasis pathogenesis are not fully understood, in recent years the contribution of dysregulated immune responses in psoriasis pathophysiology has become increasingly appreciated. In particular, the role of IL-17 producing Th17 cells has been identified as a central driving force in psoriatic inflammation, and the recent development of monoclonal antibodies targeting IL-17, and the Th17 polarising cytokine IL-23, have shown considerable clinical efficacy in severe cases of psoriasis^5,51^. Other pro-inflammatory cytokines such as IFNγ and GM-CSF have been described to contribute to the pro-inflammatory loop in psoriasis, while IL-22 directly stimulates keratinocyte proliferation^52–55^. Thus, the immune cells of principal significance in psoriasis can be identified as T cells, the primary producers of the pro-inflammatory cytokines which drive psoriatic inflammation, and DC which initiate and shape T cell immune responses. We provide data illustrating that carnosol and curcumin can maintain DC in an immature state which, in turn, impacts on antigen presentation, cytokine production and activation of adaptive T cell responses. We also demonstrate that both compounds induce robust expression of HO-1 in primary human DC and the inhibitory effects observed are, at least, partially mediated by HO-1 activity and its reaction product, CO. In addition to anti-inflammatory effects in DC, our data also shows that curcumin robustly inhibits proliferation, pro-inflammatory cytokines and poly-functionality of T cells from psoriasis patient PBMC.

The anti-inflammatory and cytoprotective properties of the heme-oxygenase system have been observed in psoriasis, with multiple reports demonstrating that increased HO-1 expression is protective in animal models of the disease. Furthermore, HO-1 is believed to contribute in part to the therapeutic effects of some existing psoriasis therapies^11–16^. However, there remains a need to identify safer and better tolerated alternatives to currently available HO-1 inducers before its established anti-oxidant and anti-inflammatory properties can be harnessed to treat psoriasis. Carnosol and curcumin are two plant-derived polyphenols which have been shown to upregulate HO-1 expression and exhibit anti-inflammatory properties^24–27^. Curcumin is known to induce HO-1 expression via Nrf2 activation and numerous studies have reported that the protective anti-inflammatory and anti-oxidant effects of curcumin are attenuated in NRF2 knockout mice^56–59^. Less is known regarding HO-1 expression by carnosol, however, it has been reported that carnosol upregulates HO-1 expression via the PI3K/AKT pathway leading to NRF2 activation in PC12 cells^27^ and HepG2 cells^60^. Curcumin has previously been shown to improve psoriasis symptoms in three small clinical trials^32–34^; however, aside from a study by Antiga *et al*.^32^ reporting a reduction in serum IL-22 concentrations in psoriasis patients receiving oral curcumin treatment, there have been no reports highlighting the effects of curcumin on human T cell cytokines. Furthermore, and to our knowledge, the effects of carnosol on human immune cells have not been reported on nor has it been assessed in the context of psoriasis.

Curcumin has previously been shown to reduce expression of DC maturation markers in murine BMDC^28^ and human monocyte-derived DC^29,37^. Consistent with this, we observed a dose-dependent reduction in expression of the co-stimulatory receptors, CD80 and CD86, and the maturation markers, CD40 and CD83, upon curcumin treatment of LPS-stimulated DC. Reduced expression of these markers was also observed in carnosol-treated DC and these effects were, at least, partially reversed by inclusion of the CO scavenger, hemoglobin. The immature phenotype observed in carnosol- and curcumin-treated DC is supported by functional analyses which demonstrated that treated DC possess a greater capacity for antigen capture and reduced pro-inflammatory responses. There have been mixed reports regarding the effects of curcumin on antigen uptake with Shirley *et al*. reporting a decrease in antigen uptake after curcumin treatment^29^, while Kim *et al*. demonstrated an increase in phagocytosis^28^. There have been no studies, to date, examining the effect of carnosol on antigen capture. In our hands, antigen uptake and processing of the model antigen, DQ-Ova, was significantly increased in LPS-treated DC which were pre-treated with carnosol or curcumin and reflected that of immature DC. Furthermore, we demonstrate that the increased antigen uptake observed upon carnosol or curcumin treatment is dependent on HO-1 activity as addition of the HO-1 enzymatic inhibitor, SnPP, almost completely attenuated this effect. As mentioned previously, HO-1 catalyses the conversion of free heme to the linear tetrapyrrole, biliverdin, with the concomitant release of CO. In contrast to carnosol or curcumin, biliverdin treatment did not increase DC antigen uptake suggesting that the effects observed are likely due to CO release. Indeed, CO has previously been reported to increase phagocytosis in murine macrophages^61,62^. Furthermore, CO has also been demonstrated to inhibit DC maturation and antigen presentation to T cells and to inhibit T cell proliferation^21,46–48^. In support of this hypothesis, DQ-Ova uptake by carnosol treated DC was significantly reduced in the presence of the CO scavenger, hemoglobin. A modest decrease in DQ-Ova uptake in curcumin treated DC in the presence of hemoglobin was also observed. Assays directly measuring HO-1 activity in primary DC are, however, required to fully validate these findings.

IL-12 and IL-23 are important cytokines which polarise T cells into Th1 and Th17 effector subsets, respectively, and have been associated with psoriasis pathology^6,51^. Expression of both of these cytokines by human DC was reduced to almost undetectable levels with carnosol and curcumin treatment. IL-1β is involved in the differentiation of Th17 cells^44^, the primary pathogenic T cell in psoriasis, and a reduction in expression of its pro-form was also observed. Interestingly, despite a report from Chauveau *et al*. that HO-1 induction conserves IL-10 expression in DC^18^, we observed a decrease in IL-10 with both compounds. However, our results are in agreement with previous studies that have shown reductions of both IL-12 and IL-10 in curcumin-treated cells^28,29^. Interestingly, similar to that seen in DC maturation, the reduction of IL-23 by carnosol and curcumin was reversed in the presence of the CO scavenger hemoglobin. We did observe an increase in HO-1 expression upon hemoglobin treatment suggesting there is some level of heme release over time, however this is not enough to completely abrogate the effect of CO scavenging by hemoglobin.

One potential mechanism through which the compounds may be acting is via inhibition of MAP kinase activation. MAPKs are major regulators of DC function and CoPP induced HO-1 has been reported to inhibit MAPK signalling in murine DC and endothelial cells^35,63^. Both carnosol and curcumin were found to reduce MEK and ERK activation, and a modest reduction in p38 activation was also observed. Furthermore, this reduction of MAPK activation by carnosol and curcumin was found to be dependent on the activity of HO-1, as the addition of the HO-1 inhibitor SnPP blocked this effect. SnPP also attenuated some of the anti-inflammatory effects of carnosol and curcumin on DC stimulated with LPS, including reductions in maturation marker expression and pro-inflammatory cytokine production. While neither carnosol nor curcumin had any effect of the degradation of the NFκB inhibitor, IκB, the LPS-induced translocation of p65 to the nucleus was inhibited with curcumin treatment suggesting that the two compounds may differentially affect NFκB. Further study is required to confirm in more detail the precise mechanisms underlying these effects.

Having determined that carnosol- and curcumin-treated DC display a tolerogenic phenotype, with reduced expression of co-stimulatory receptors and pro-inflammatory cytokines, we examined whether these effects would result in a reduced capacity to activate CD4^+^ T cells. Using a mixed lymphocyte reaction, we quantified the allogeneic responses of recipient to donor cells and found that carnosol- and curcumin-treated DC effectively suppressed T cell proliferation and IFNγ production. Our data therefore supports previous studies demonstrating reduced proliferation of CD4^+^ T cells cultured with curcumin-treated DC^28,29,37^ and reports, for the first time, the same effect for carnosol-treated DC.

Finally, in addition to our extensive analysis of the effects of carnosol and curcumin on the phenotype and function of human DC, we examined whether they would also possess anti-inflammatory properties within PBMC from psoriasis patients which are known to express elevated levels of pro-inflammatory cytokines^64^. In particular, we wished to assess the potential of these polyphenols to inhibit proliferation and pro-inflammatory cytokine production in T cells. Our results demonstrate that curcumin effectively inhibited proliferation and production of IFNγ, IL-17, GM-CSF and IL-22 by T cells in *ex-vivo* stimulated psoriasis PBMC. A significant reduction in the expansion of γδ T cells was also observed in curcumin-treated psoriasis PBMC, which may be of relevance in light of recent reports of the contribution of IL-17 producing γδ T cells in murine models of psoriasis^49,50^. We also report for the first time that curcumin effectively reduced T cell poly-functionality as shown in our SPICE analysis of psoriasis PBMC. Unlike curcumin, carnosol treatment had no significant effects on T cell proliferation, cytokine production or poly-functionality in psoriasis PBMC, indicating that carnosol’s anti-inflammatory effects are more potent in DC rather than PBMC.

In conclusion, our data illustrates that the plant-derived polyphenols, carnosol and curcumin, act as powerful immune-modulators by maintaining DC in an immature and tolerogenic phenotype with significantly reduced pro-inflammatory responses, with relevance to the pathophysiology of psoriasis. Moreover, we have characterised the anti-inflammatory properties of carnosol in human DC for the first time. We provide evidence that at least some of the effects of carnosol and curcumin treatment in human DC are mediated by HO-1 activity, although further investigation is warranted to establish the exact mechanism of action of these two compounds. Furthermore, curcumin also displayed a significant capacity to limit harmful T cell-mediated immune responses in *ex-vivo* psoriasis PBMC. The present study supports previously published clinical trials of curcumin in psoriasis, and future research into the immunomodulatory effects of carnosol and curcumin. The use of these molecules as oral therapies is very much dependent on their bioavailability given that they are highly insoluble compounds. A recent clinical trial has demonstrated that oral delivery of Meriva (a lecithin based delivery system of curcumin) was effective as an adjuvant therapy for the treatment of psoriasis and was shown to significantly reduce serum levels of IL-22 and PASI scores in patients with mild-moderate disease^32^. It will be interesting to determine if orally available carnosol shows a similar efficacy in future trials.

## Electronic supplementary material

Supplementary Information
