# Supplementary material for: Naturally derived Heme-Oxygenase 1 inducers attenuate inflammatory responses in human dendritic cells and T cells: relevance for psoriasis treatment
Source: Sci Rep. 2018 Jul 6;8:10287. doi: 10.1038/s41598-018-28488-6 (PMC6035209; doi:10.1038/s41598-018-28488-6)
Supplement: Supplementary file 1 — Supplementary Information [file 41598_2018_28488_MOESM1_ESM.docx]

**Supplementary information**

**Naturally derived Heme-Oxygenase 1 inducers attenuate inflammatory responses in human dendritic cells and T cells: relevance for psoriasis treatment.**

Nicole Campbell^1^, Hannah Fitzgerald^1^, Anna Malara^2^, Roisin Hambly^2^, Cheryl Sweeney^2^, Brian Kirby^2^, Jean Fletcher^1^ and Aisling Dunne^1^*

^1^School of Biochemistry & Immunology and School of Medicine, Trinity Biomedical Sciences Institute, Trinity College Dublin, the University of Dublin, Dublin 2, Ireland.

^2^Department of Dermatology, St. Vincent’s University Hospital, Dublin 4, Ireland.

*Address correspondence to: [aidunne@tcd.ie](mailto:aidunne@tcd.ie), Ph: +353-1-8962437, Fax: +353-1-6772400.

**Supplemental Figure 1. Carnosol and curcumin do not activate MAP Kinases in human DC.**

DC from healthy donors (n=3) were incubated with LPS (100 ng/ml), carnosol (10 µM) or curcumin (10 µM) for 15 minutes to 3 hours. The activation of the MAPKs MEK and p38, as was measured by western blot. Data shown is representative of 3 healthy donors. Full length blots are presented in Supplemental Figure 12.

**Supplemental Figure 2. Full size blots for Figure 1.**

HO-1 expression in human DC was determined by western blot. Membrane was first probed for HO-1 and then the same membrane re-probed for β-actin. Blots were developed using enhanced chemiluminescent substrate with a BioRad ChemiDoc MP system. Full scan images of the cropped exposure images used in Figure 1C **(A)** and Figure 1D **(B)** are provided alongside merged images of the exposed bands with the protein ladder to show molecular weights of the proteins.

**Supplemental Figure 3. Full size blots for Figure 4.**

Pro-IL-1β expression in human DC was determined by western blot. Membrane was first probed for Pro-IL-1β and then the same membrane re-probed for β-actin. Blots were developed using enhanced chemiluminescent substrate with a BioRad ChemiDoc MP system. Full scan images of the cropped exposure images used in Figure 4C are provided alongside merged images of the exposed bands with the protein ladder to show molecular weights of the proteins.

**Supplemental Figure 4. Full size blots for Figure 5 (P/MEK & P/ERK).**

Activation of the MAPKs MEK and ERK in human DC was determined by western blot. Phospho- and Total- proteins were run on the separate gels where possible, using the same amounts of samples in each gel. Where sample amounts were limited, membranes were first probed for phospho proteins and then the same membranes stripped & re-probed for total proteins. Blots were developed using enhanced chemiluminescent substrate with a BioRad ChemiDoc MP system. Full scan images of the cropped exposure images used in Figure 5A are provided alongside merged images of the exposed bands with the protein ladder to show molecular weights of the proteins.

**Supplemental Figure 5 Full size blots for Figure 5 (P/P38 & IκB).**

Activation of the MAPK p38 and IκB degradation in human DC was determined by western blot. Phospho- and Total- proteins were run on the separate gels where possible, using the same amounts of samples in each gel. Where sample amounts were limited, membranes were first probed for phospho proteins and then the same membranes stripped & re-probed for total proteins. Blots were developed using enhanced chemiluminescent substrate with a BioRad ChemiDoc MP system. Full scan images of the cropped exposure images used in Figure 5A are provided alongside merged images of the exposed bands with the protein ladder to show molecular weights of the proteins.

**Supplemental Figure 6. Full size confocal images for Figure 5.**

Nuclear translocation of p65 in LPS stimulated DC was assessed by confocal microscopy. Full size images of the cropped images used in Figure 5B are provided.

**Supplemental Figure 7. Full size blots for Figure 6.**

Activation of the MAPKs MEK and p38 in human DC was determined by western blot. Phospho- and Total- proteins were run on the separate gels, using the same amounts of samples in each gel. Blots were developed using enhanced chemiluminescent substrate with a BioRad ChemiDoc MP system. Full scan images of the cropped exposure images used in Figure 6A are provided alongside merged images of the exposed bands with the protein ladder to show molecular weights of the proteins.

**Supplemental Figure 8. Full size blots for Figure 7.**

HO-1 expression in human DC treated with hemoglobin for 24 hours was determined by western blot. Membrane was first probed for HO-1 and then the same membrane re-probed for β-actin. Blots were developed using enhanced chemiluminescent substrate with a BioRad ChemiDoc MP system. Full scan images of the cropped exposure images used in Figure 6F are provided alongside merged images of the exposed bands with the protein ladder to show molecular weights of the proteins.

**Supplemental Figure 9. Full size blots for Figure 9.**

HO-1 expression in human PBMC treated was determined by western blot. Membrane was first probed for HO-1 and then the same membrane re-probed for β-actin. Blots were developed using enhanced chemiluminescent substrate with a BioRad ChemiDoc MP system. Full scan images of the cropped exposure images used in Figure 8E are provided alongside merged images of the exposed bands with the protein ladder to show molecular weights of the proteins.

**Supplemental Figure 10. Flow cytometry gating strategies for DC experiments.** **(A)** For assessing DC viability and expression of maturation markers, the DC population was first selected by forward (FSC) and side scatter (SSC). Single cells were then gated on using forward scatter width and height. Finally, viable cells were selected on the basis of viability dye exclusion. **(B)** To measure apoptosis DC were stained with a fluorochrome-conjugated antibody for Annexin V and propidium iodide (PI). Debris events were selected on the basis of low FSC and SSC. Next, events which did not stain positive for Annexin V or PI were selected. Debris events were selected from this population again on the basis of low FSC and SSC and this gate was applied to the total population to select events outside (non-debris events). The Annexin V and PI gates were then set using single-stained control cells. **(C)** To measure DQ-Ova uptake, the DC population was first selected on the basis of FSC and SSC in order to exclude debris and dying cells. Single cells were then selected on the basis of FSC width and height. Finally, the DQ-Ova^+^ cell gate was drawn using control cells which were not incubated with DQ-Ova. **(D)** To measure CD4+ T cell proliferation, the CD4 T cell population was selected on the basis of FSC and SSC. CellTraceViolet fluorescence was then compared between samples by histogram.

**Supplemental Figure 11. Flow cytometry gating strategy for *ex-vivo* psoriasis PBMC analysis.**

To assess the proliferation and cytokine production by T cells in *ex-vivo* stimulated psoriasis PBMC, lymphocytes were first gated on based on forward and side scatter. Next single cells were gated on based on size to exclude doublet cells. Viable cells were selected on the basis of viability dye exclusion. Within the live cell population, the proportion of CD3^+^TCRγδ^+^ T cells was determined. As the CD4 receptor is often downregulated during restimulation with PMA and ionomycin, CD3^+^CD8^-^ were selected for cytokine and proliferation analysis, comprising primarily of CD4^+^ T cells and the small percentage of γδ T cells. Representative dot plots for Ki67 and cytokine staining in the CD3^+^CD8^-^ population are shown; cytokine gates were set with the aid of an unstimulated control, i.e. PBMC from a healthy donor incubated without PMA or ionomycin, in the presence of brefeldin A.

**Supplemental Figure 12. Full size blots for Supplementary Figure 1.**

Activation of the MAPKs MEK and p38 in human DC was determined by western blot. Phospho- and Total- proteins were run on the separate gels, using the same amounts of samples in each gel. Blots were developed using enhanced chemiluminescent substrate with a BioRad ChemiDoc MP system. Full scan images of the cropped exposure images used in Supplemental Figure 1 are provided alongside merged images of the exposed bands with the protein ladder to show molecular weights of the proteins.

**Supplemental Figure 1**

**
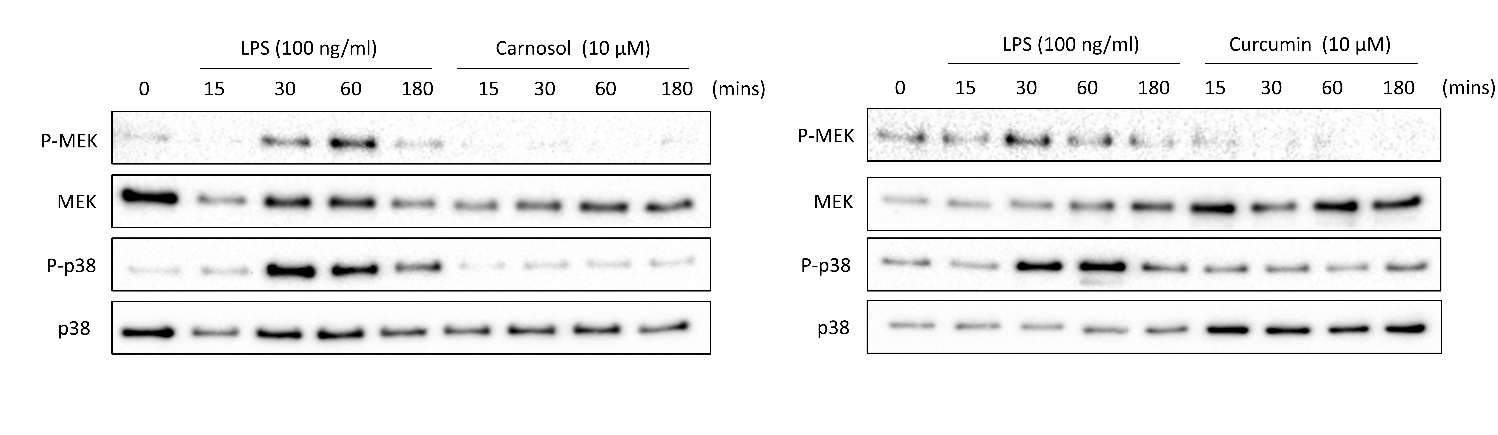
**

**Supplemental Figure 2**

**
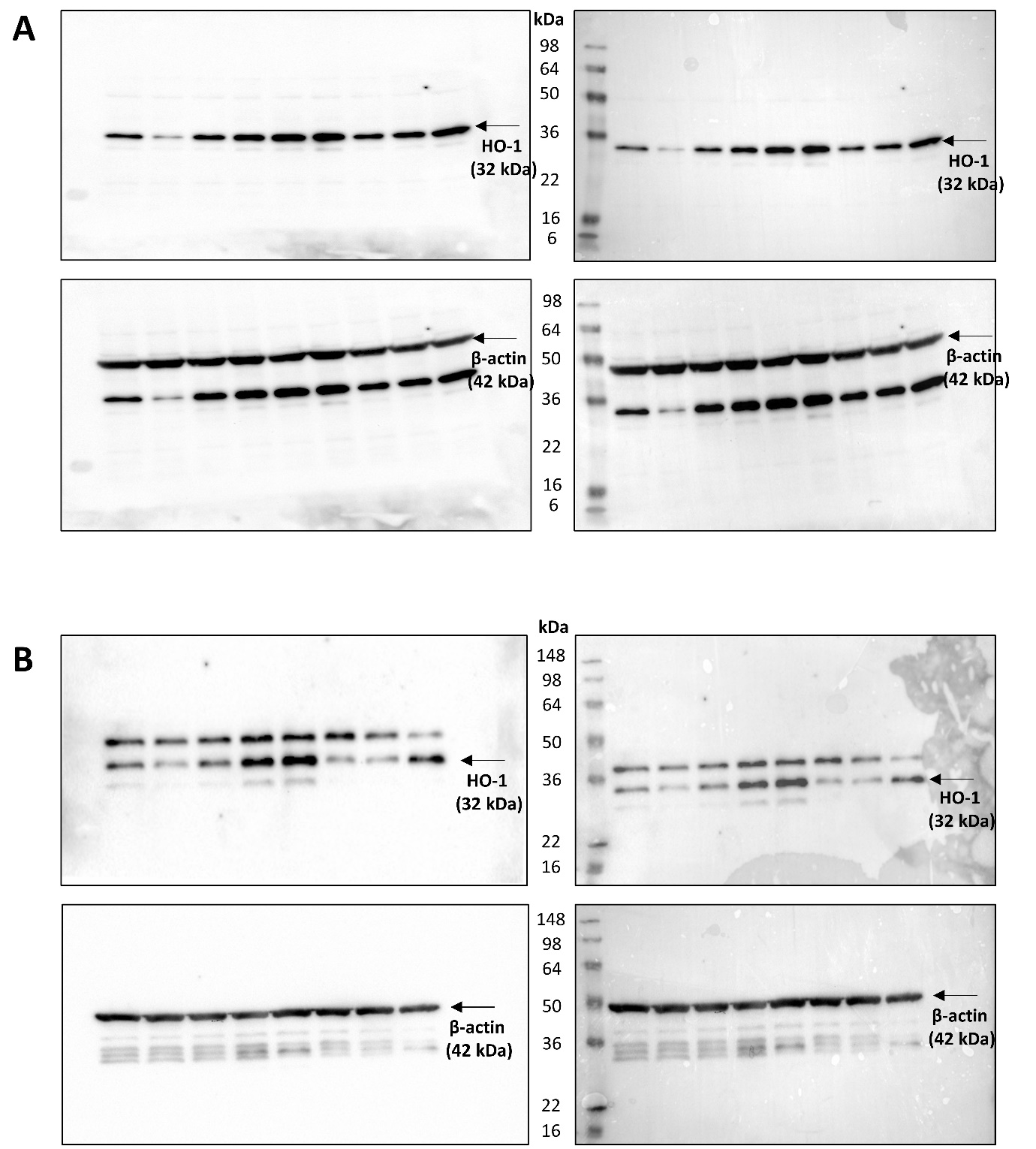
**

**Supplemental Figure 3**

**
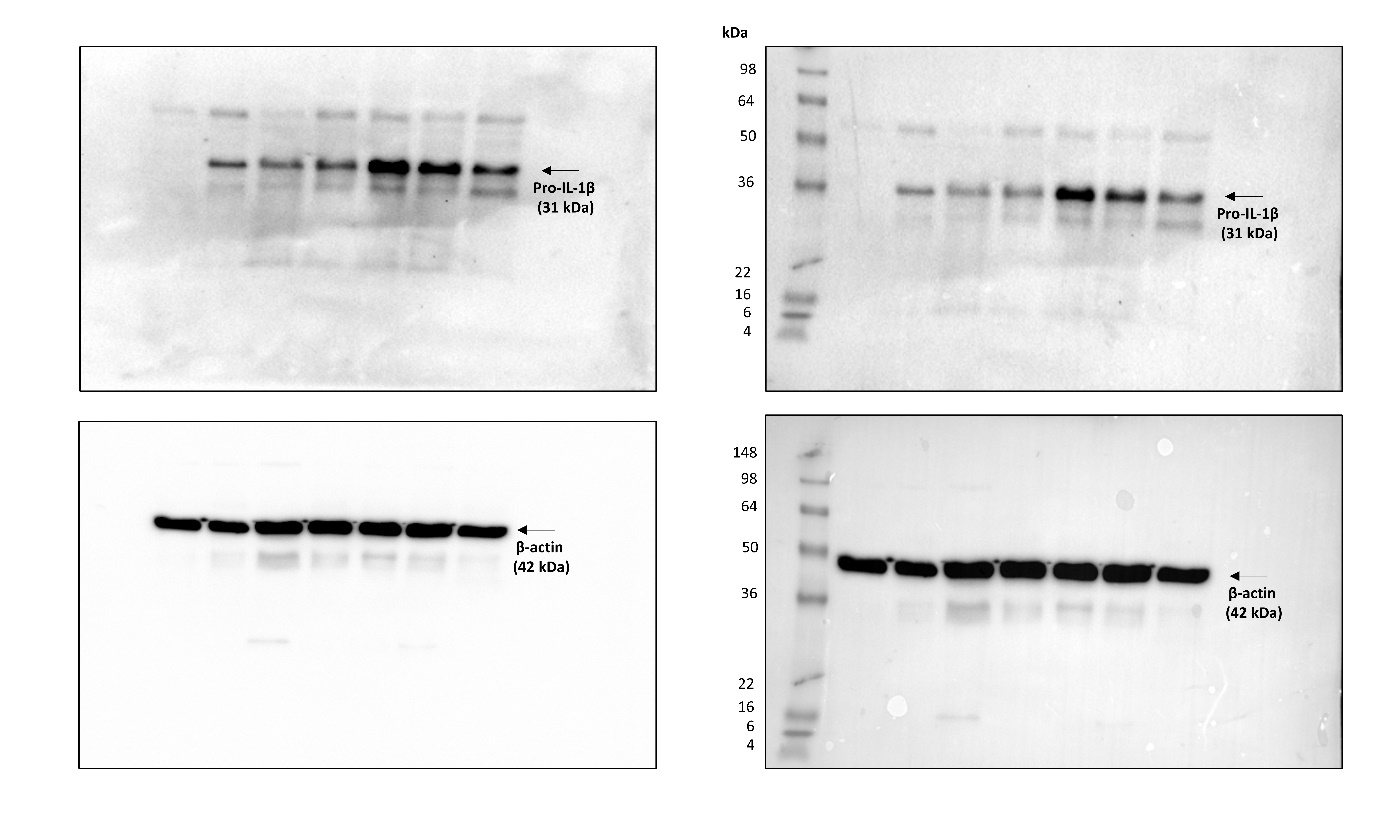
**

**Supplemental Figure 4**

**
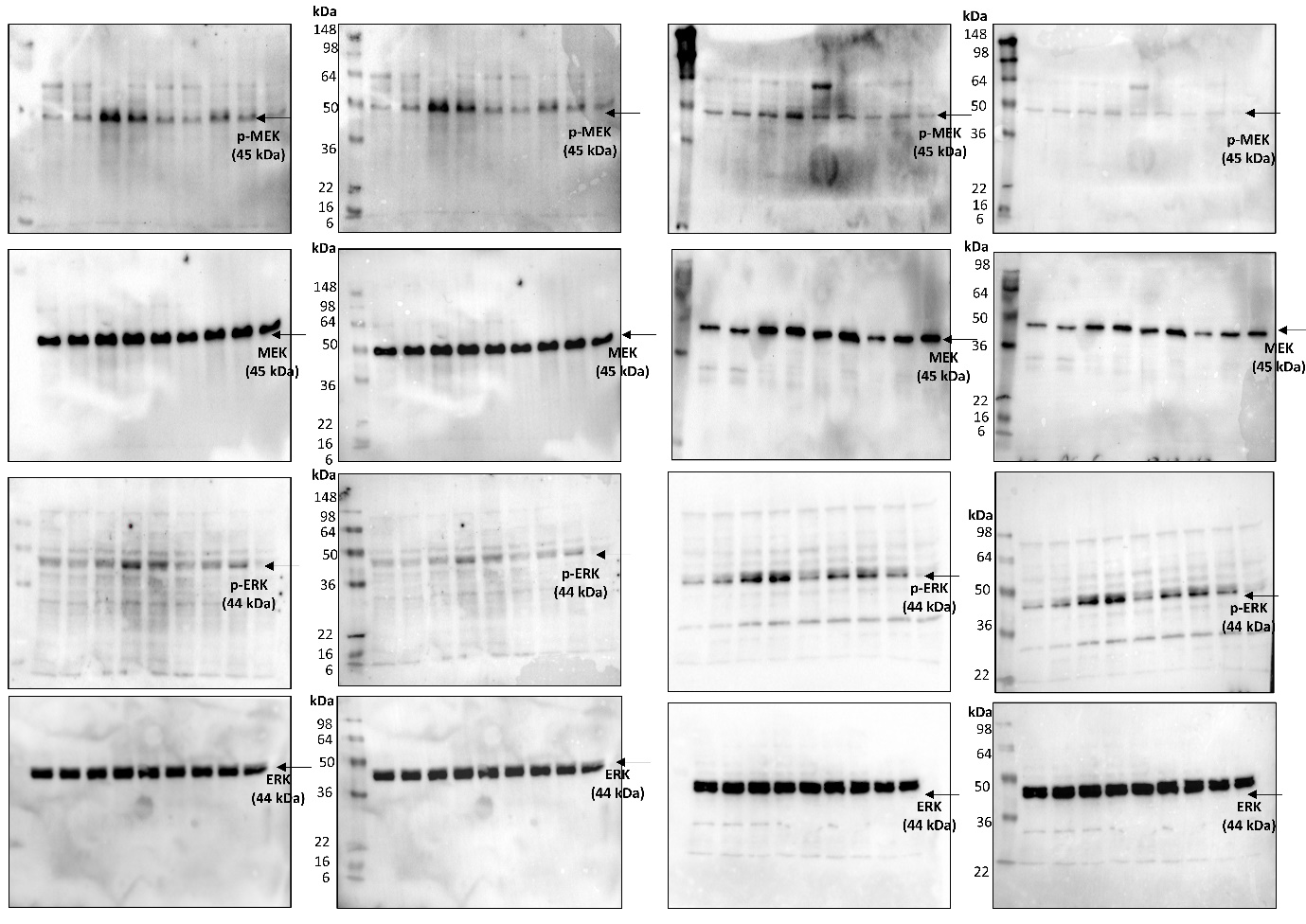
**

**Supplemental Figure 5**

**
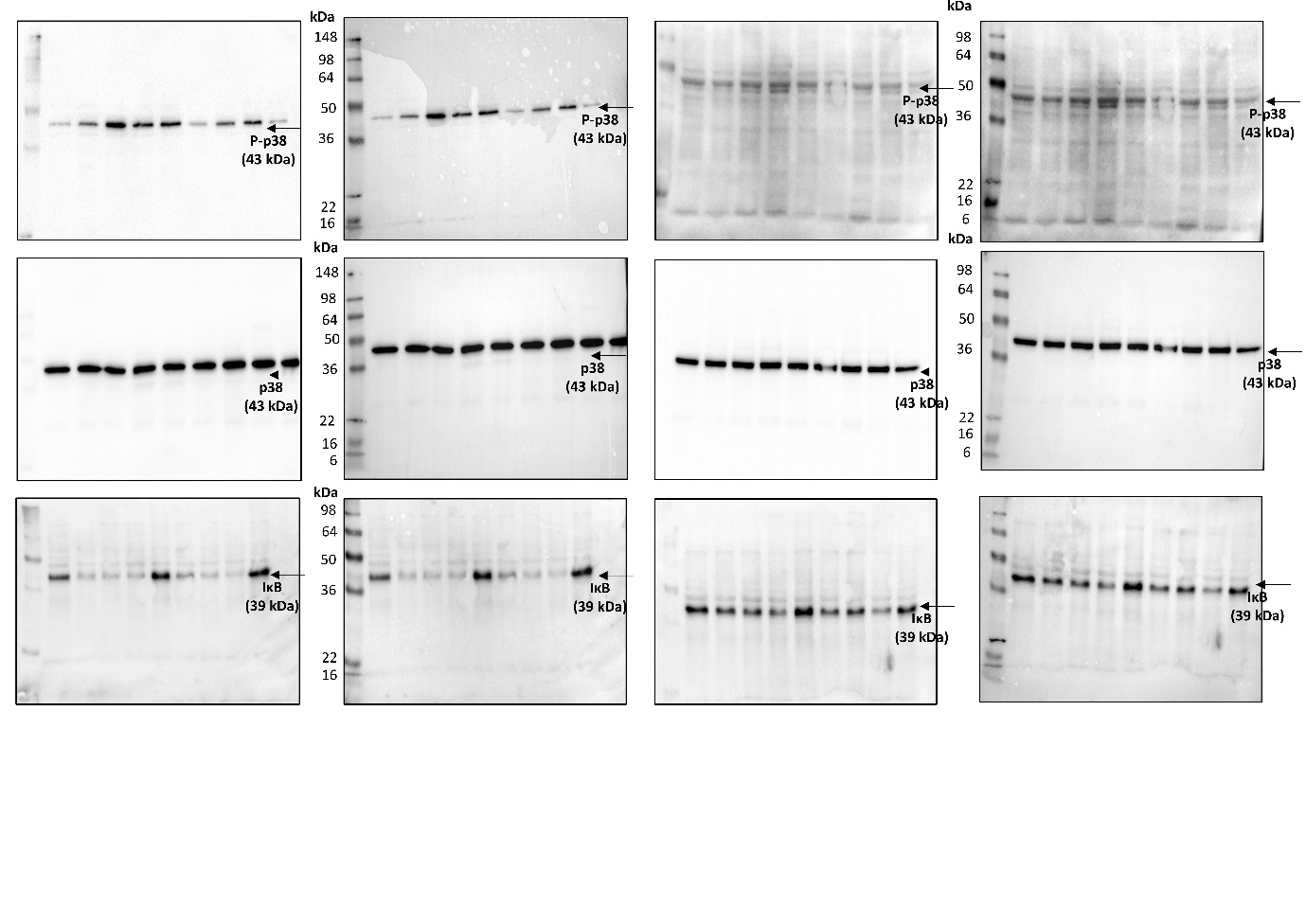
**

**Supplemental Figure 6**

**
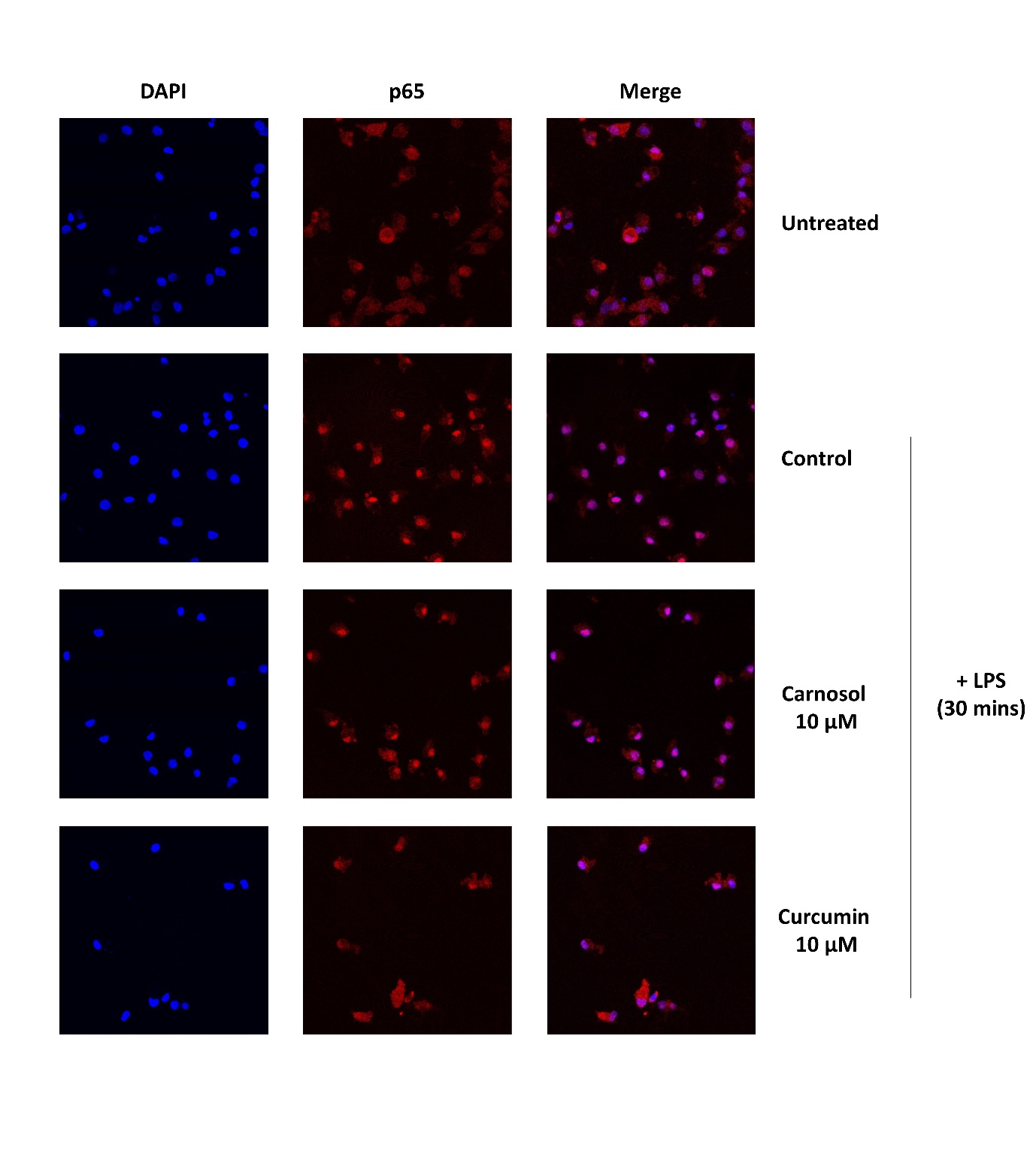
**

**Supplemental Figure 7**

**
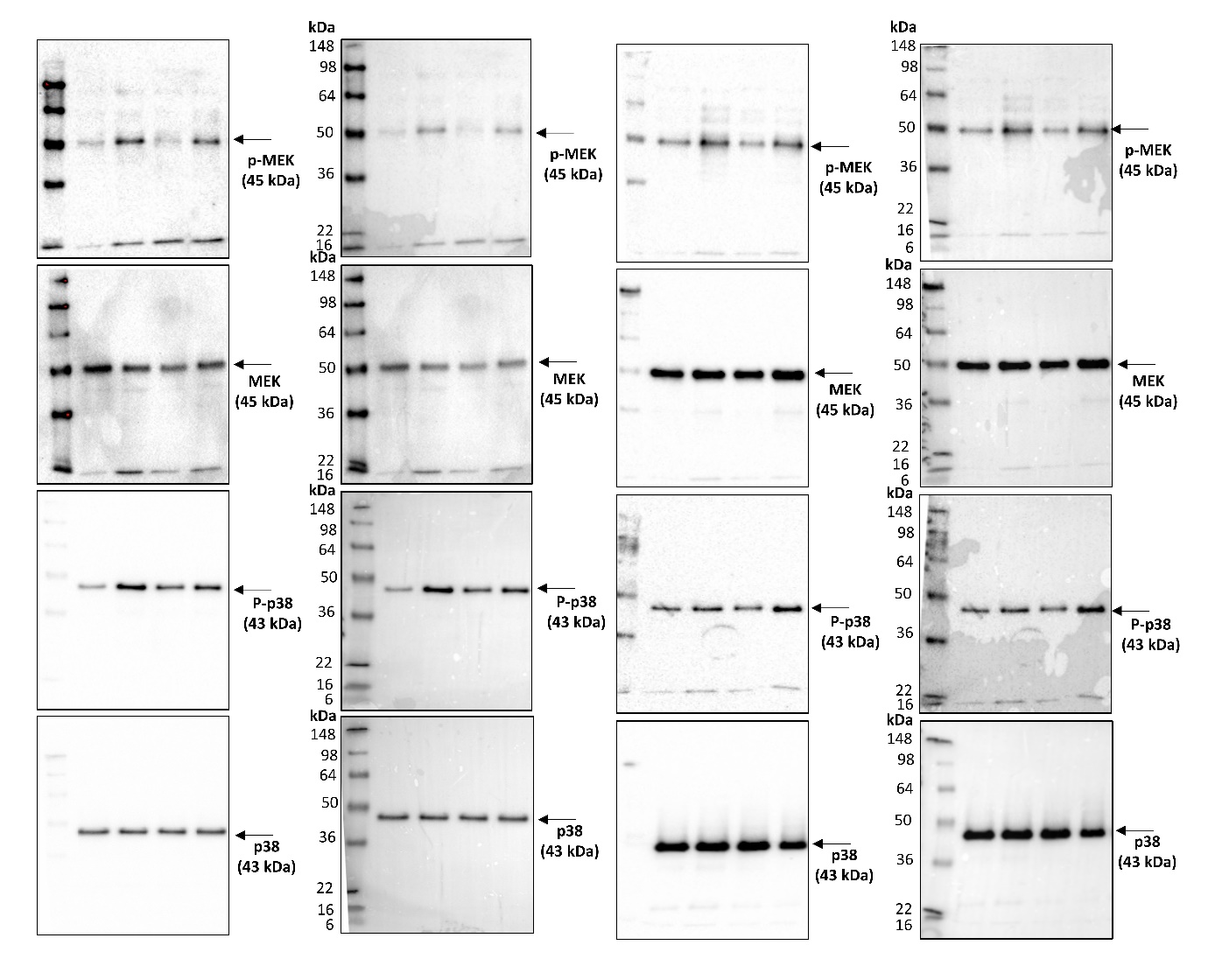
**

**Supplemental Figure 8**

**
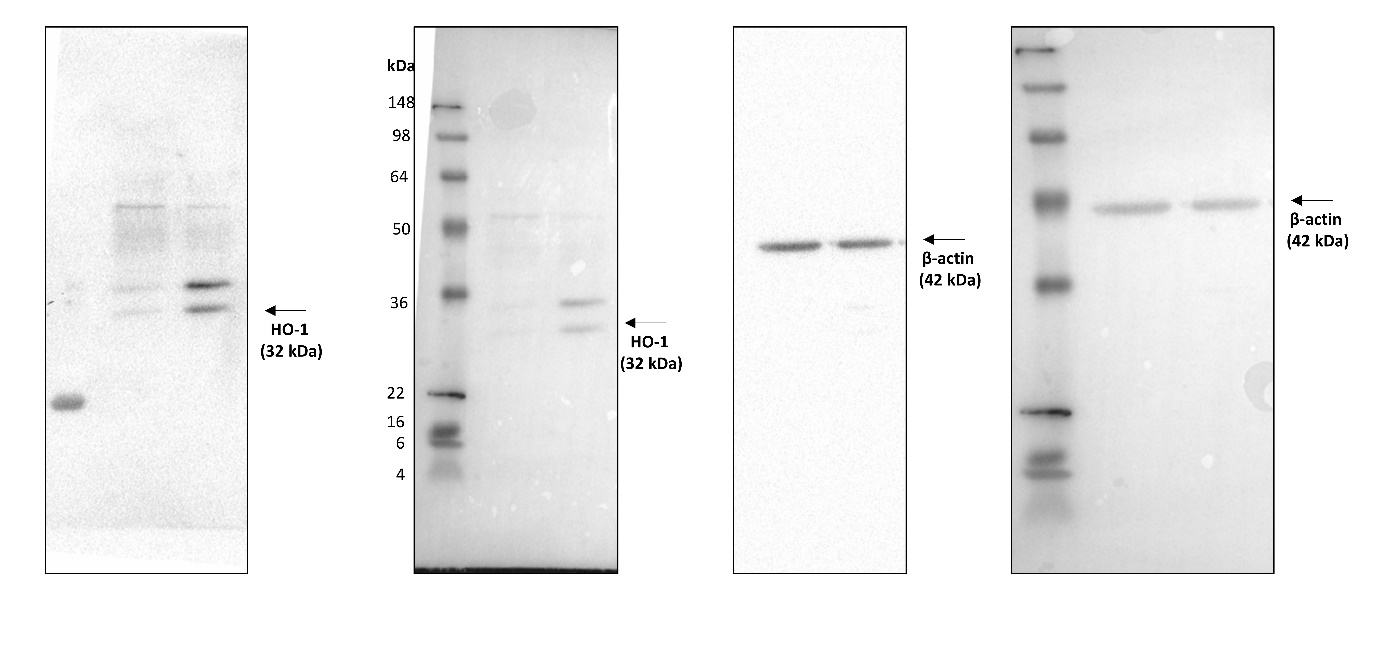
**

**Supplemental Figure 9**

**
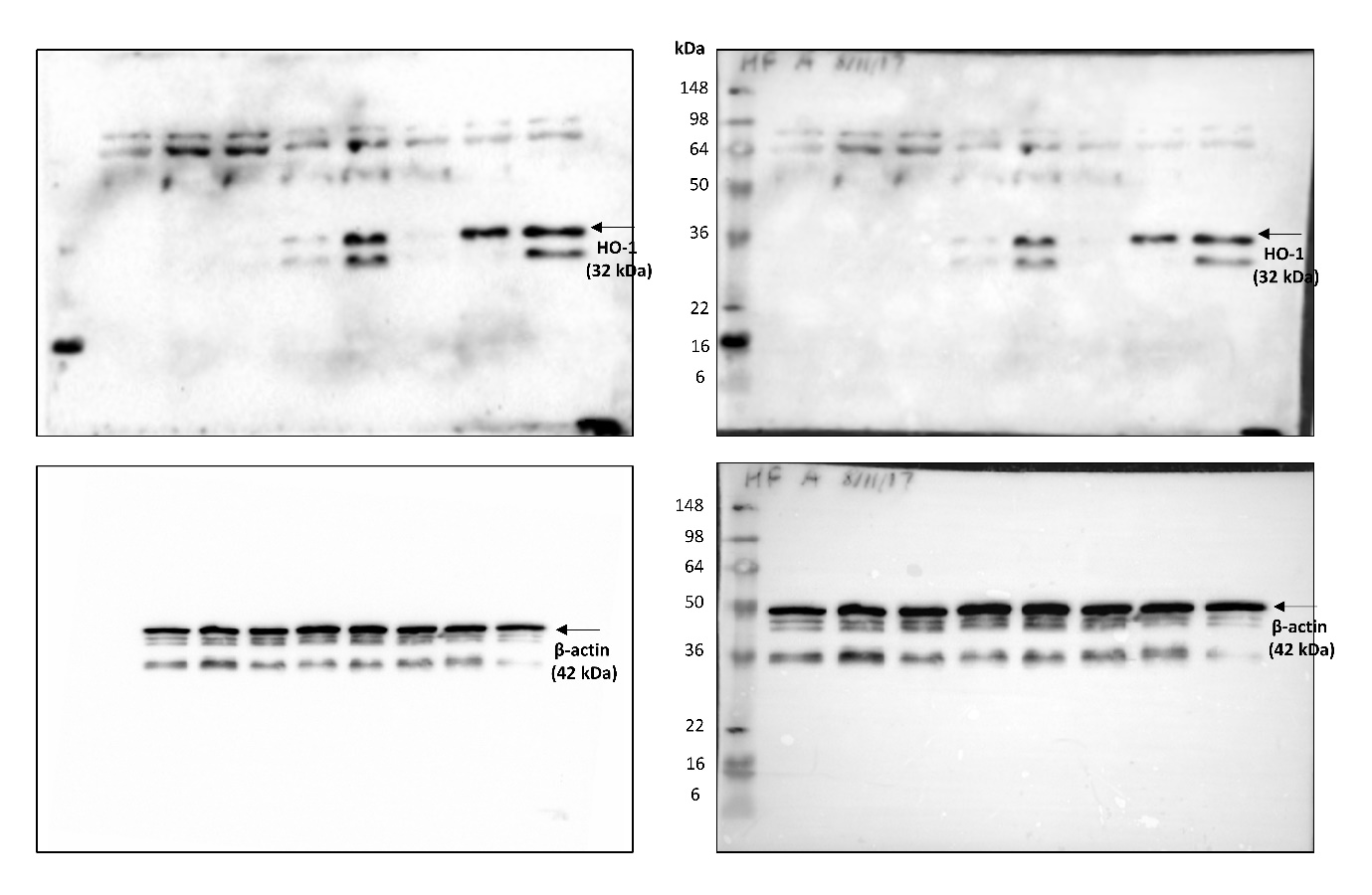
**

**Supplemental Figure 10**

**
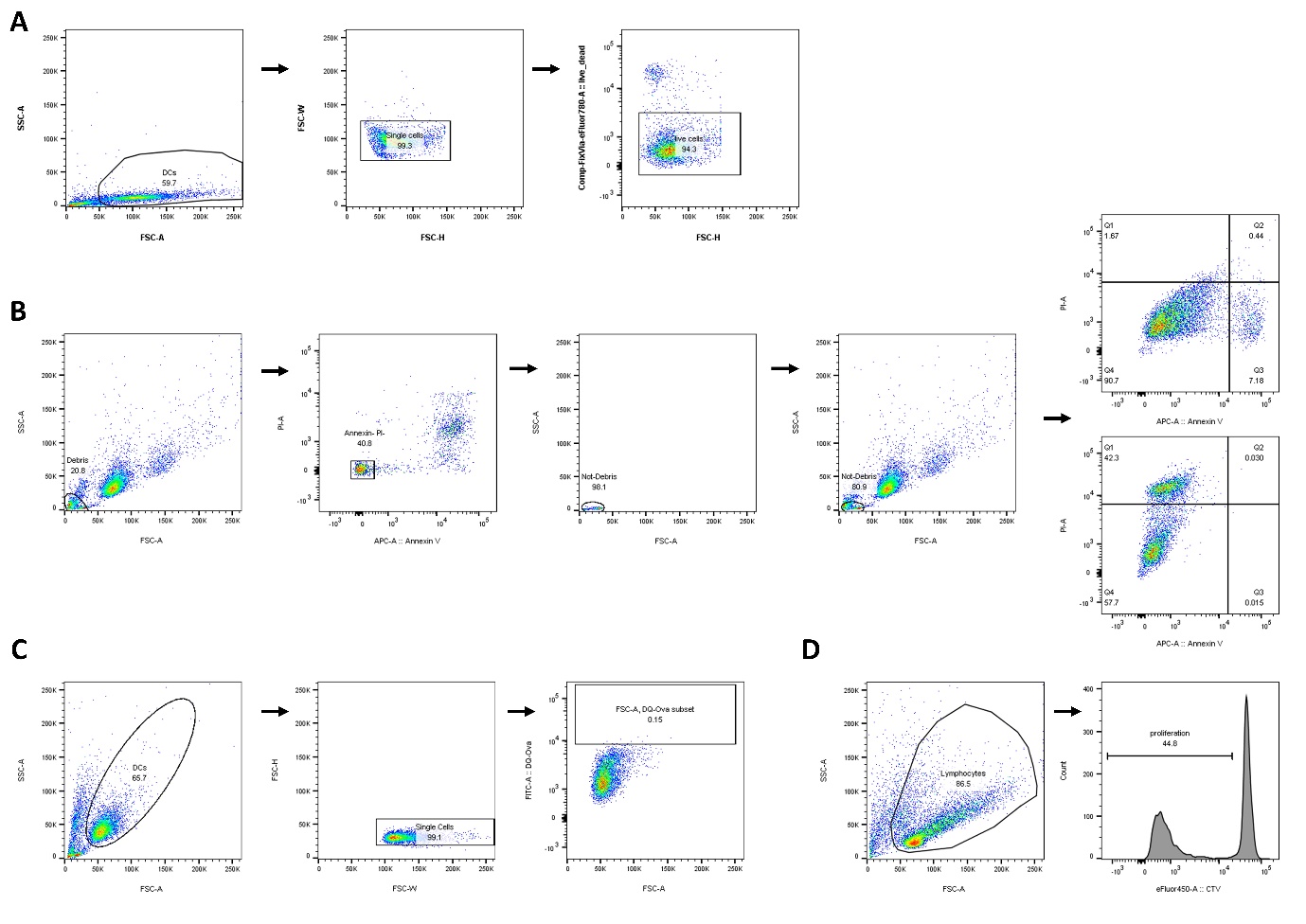
**

**Supplemental Figure 11**

**
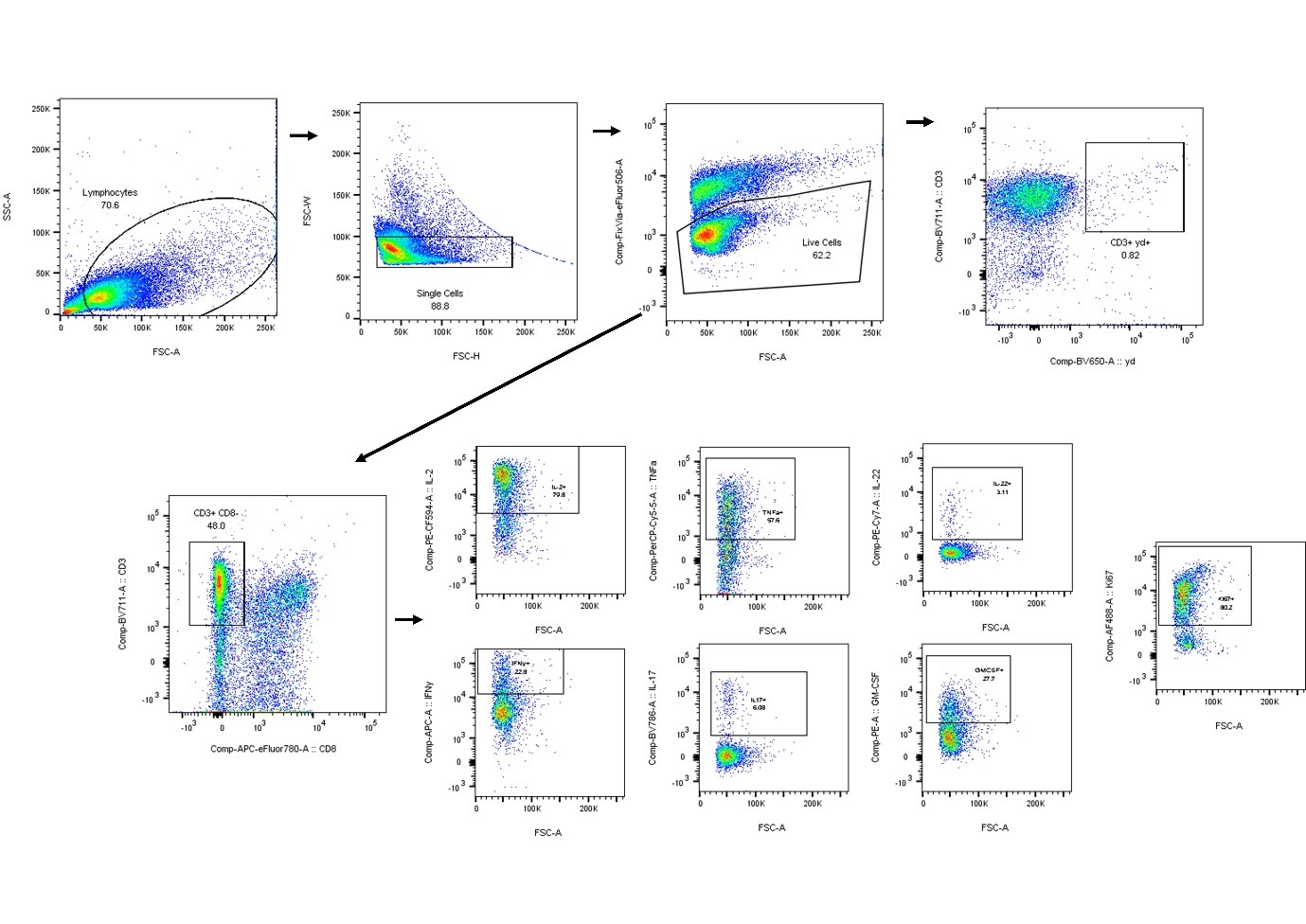
**

**Supplemental Figure 12**

**
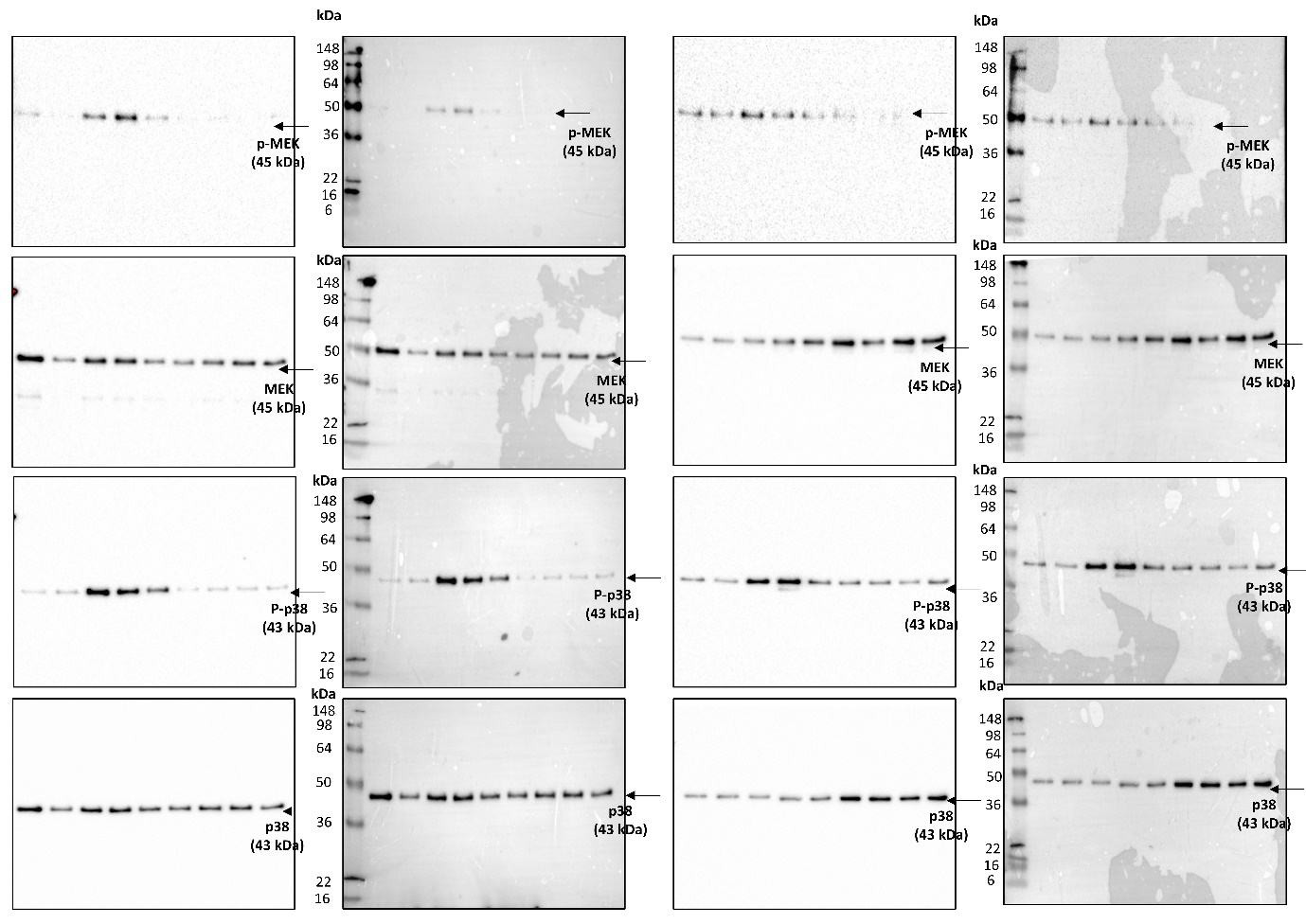
**
